# A Quantitative Proteomic Approach Explores the Possible Mechanisms by Which the Small Molecule Stemazole Promotes the Survival of Human Neural Stem Cells

**DOI:** 10.3390/brainsci12060690

**Published:** 2022-05-25

**Authors:** Mingzhu Chen, Yizi Zhu, Huajun Li, Yubo Zhang, Mei Han

**Affiliations:** Key Laboratory of Radiopharmaceuticals, College of Chemistry, Beijing Normal University, Ministry of Education, Beijing 100875, China; mingzhucc@126.com (M.C.); zhuyizi1112@163.com (Y.Z.); lihuajun726@foxmail.com (H.L.); zhangyb1995@163.com (Y.Z.)

**Keywords:** quantitative proteomics, human neural stem cells, stemazole, anti-apoptosis, molecular mechanisms

## Abstract

Neurodegenerative disorders have become a serious healthcare problem worldwide and there is no efficacious cure. However, regulating the fate of stem cells is an effective way to treat these neurological diseases. In previous work, stemazole was reported to maintain the survival of human neural stem cells in the absence of growth factors and to have therapeutic effects on neurodegenerative diseases. However, although it is a promising small molecule, the molecular mechanisms against apoptosis are ambiguous. In this study, tandem mass tag (TMT)-based proteomics were performed to obtain whole protein expression profiles of human neural stem cells in different groups under extreme conditions. Bioinformatics analysis based on protein–protein interaction (PPI) network construction, gene ontology (GO) and the Kyoto Encyclopaedia of Genes and Genomes (KEGG) pathway enrichment analysis were adopted to explore crucial proteins and possible pharmacological mechanisms. A total of 77 differentially expressed proteins were identified, comprising 38 upregulated proteins and 39 downregulated proteins. Combined with a diseases database of Alzheimer’s disease (AD), caspase-2 (CASP2), PKA C-alpha (PRKACA), fibronectin (FN1), large neutral amino acid transporter small subunit 1 (SLC7A5), which are involved in cell proliferation and apoptosis, this was further validated by enzyme activity assay and molecular docking, and regarded as putative targets regulated by stemazole. The present results give an insight into this small molecule and a better understanding for further elucidating the underlying mechanisms in the treatment of stem cells and neurodegenerative diseases.

## 1. Introduction

Stem cells, unspecialized cells existing in organisms, have the capacity for self-renewal and differentiation into multiple cell types. They are located in special microenvironments, which are also called niches, and they regulate cell developmental processes and fate by communicating through mechanical forces [1,2]. Cell-based therapies have emerged as necessary based on their features. The capability of stem cells to be induced and replace damaged, destroyed cells or tissues gives them great potential. Diabetes [3], osteoarthritis [4], strokes [5] and neurodegenerative diseases [6,7] all benefit from this alternative therapy.

Neurodegenerative diseases, which are chronic progressive diseases characterized by the loss of selective neuronal populations, are observed to worsen with age and impact many regions; in addition, these diseases have risen sharply, posing a serious threat to the physical and mental health of the elderly [8,9,10]. Alzheimer’s disease (AD) is a typical disease that causes memory loss, behaviour disorders and cognitive impairment. According to the World Alzheimer Report 2021, it is predicted that approximately 152 million people will be living with dementia by 2050, and the number of individuals aged 65 or older is expected to reach 12.7 million. AD is already the fifth leading cause of death in the world [11,12]. Parkinson’s disease (PD), the second most common neurodegenerative disorder of the central nervous system after AD, leads to shaking, rigidity and difficulty with walking, balance and coordination. It might be the fastest growing neurological disease worldwide. By 2015, the number of people with Parkinson’s disease was over 6 million, and this number is predicted to double to more than 12 million by 2040 [13]. Unfortunately, there is no standard treatment or effective drugs against these neurological disorders and treatments can only to delay progression [14].

Stem cell replacement therapy seems to be hugely superior to other methods as it replaces deteriorated cells and improves cognitive function from the source [15,16,17]. However, the therapy still involves many obstacles, such as ethical concerns and the immunological tolerance between exogenous stem cells and the patient’s body [2,18]. Thus, it is urgent to find innovative approaches such as small molecular drugs that regulate endogenous stem cells to proliferate and directionally differentiate to become substitutes for injured cells.

Stemazole, which emerged from high-throughput screening of tens of thousands of compounds, is a small molecule and has proficiency in preventing multiple kinds of stem cells, including human neural stem cells (hNSCs), embryonic stem cells, pancreatic stem/progenitor cells and cardiac stem/progenitor cells, from apoptosis in conditions of injury or cell deficiencies [19,20]. In addition, previous in vivo studies have demonstrated the therapeutic effects and symptom improvements of stemazole in preclinical rodent models of neurodegenerative diseases, including AD and PD, and the pharmacokinetic study showed that stemazole has suitable oral bioavailability [21,22,23]. Network pharmacology was conducted to reveal the mechanism involved in the improvement of AD and PD symptoms and found five critical targets, including caspase-3 (CASP3), caspase-8 (CASP8), mitogen-activated protein kinase 8 (MAPK8), mitogen-activated protein kinase 14 (MAPK14) and RAC-alpha serine/threonine-protein kinase (AKT1) [24]. Interestingly, further research found that the addition of stemazole led to hNSC cytotoxicity under supplementation sufficiency. However, the molecular mechanisms of how this small molecule works remain unclear. To obtain a complete understanding and decode the complicated mechanisms of stemazole regarding different feedbacks of hNSCs in two conditions, proteomics research was conducted.

Proteomics is a newly developed tool for the simultaneous detection of thousands of proteins and an alternative to the conventional molecular biology approach. Through the difference in protein expression profiles and further bioinformatics methods in different groups, the involved pathways or even precise regulatory targets could be identified [25,26,27]. TMT labelling is one of the most widely used quantitative proteomics techniques [28,29]. Therefore, the first proteomics study was performed under normal conditions [30]. Notable upregulated protein-cytochrome P450 and downregulated proteins related to mitochondrial respiratory chain enzymes were screened out, which indicated that stemazole affected the mitochondrial function of hNSCs with a healthy status.

In this work, firstly, a TMT-labelled proteomics investigation was conducted to further obtain the difference in protein abundance profiles with or without stemazole under extreme conditions. Following this, bioinformatics like PPI network, GO, and KEGG analysis were performed to further clarify the key proteins, relative biological processes and pathways regulated by stemazole. Furthermore, some essential proteins involved in cell proliferation, apoptosis and neurodegenerative diseases were focused on and verified by functional assay and molecular docking. We hope that this work may provide a better understanding of this small molecule and the mechanism in human neural stem cells and neurodegenerative diseases.

## 2. Materials and Methods

### 2.1. Cell Culture and Treatment

Human neutral stem cells were obtained from the Stem Cell Centre of Peking University (Beijing, China). These cells were cultured in DMEM/F12 medium (Sigma, San Francisco, CA, USA) supplemented with 0.02% epidermal growth factor (EGF), 0.02% fibroblast growth factor (FGF), 2% B27 supplements, 100 U/mL streptomycin and 100 U/mL penicillin (Gibco, Carlsbad, CA, USA) in a humidified atmosphere with 5% CO_2_ at 37 °C. hNSCs were cultured in basic DMEM/F12 medium with removal of growth factors overnight before treatment.

Stemazole (purity > 98%) was synthesized by the Key Laboratory of Radiopharmaceuticals of Beijing Normal University (Beijing, China). The powder was dissolved in DMEM/F12 medium and gradient diluted to reach the target concentration. Cell viability was quantified by CellTiter-Glo^®^ (Promega, WI, USA).

### 2.2. Total Protein Extraction and Preparation of Proteomic Samples

According to the dose and time response assays (Figure S1), we chose 40 μM and 4 days of treatment with stemazole to investigate the protein expression profiles on hNSCs. The methods of protein extraction have been described in previous studies [30,31]. Briefly, the cells were inoculated in 6-well plates in the absence of growth factors, and with or without treatment of stemazole in 40 μM for 4 days. Each group has three biological independent replicates. Subsequently, the six samples were collected and centrifuged at 1000× *g* for 5 min. The precipitate was washed with precooled PBS solution (HyClone, Logan, UT, USA) three times. Cells were collected, frozen in liquid nitrogen quickly and saved at −80 °C. The samples were lysed with DB lysis buffer (100 mM TEAB, 8 M urea, pH = 8.5). After ultrasonication and centrifugation, 10 mM DTT was mixed for 1 h at 56 °C, and then the samples were alkylated with iodoacetamide for 1 h in the dark.

### 2.3. Protein Quality Test

BSA standard protein solution was prepared to quantify the protein concentration according to the instructions of the Bradford protein quantitative kit (Baoruijie, Beijing, China). Briefly, by detecting the absorbance of 595 nm of samples and drawing the standard curve, the protein concentration was determined. Protein samples (20 μg) were loaded for 12% SDS–PAGE (concentrated gel was set at 80 V for 20 min and 120 V for 90 min in the separation gel). After that, Coomassie brilliant blue R-250 was added (Bio-Rad, Hercules, CA, USA), and when bands were visualized clearly, decolouring was performed.

### 2.4. TMT Peptides Labelling and Separation of Fractions

The protein samples were added to 100 μL of DB dissolution buffer, trypsin and 100 mM TEAB buffer, and digested at 37 °C for 4 h. Following this, the samples were mixed with formic acid and centrifuged at 12,000× *g* for 5 min. The supernatant was loaded onto a C18 desalting column and washed 3 times with washing buffer (0.1% formic acid and 3% acetonitrile) and elution buffer (0.1% formic acid and 70% acetonitrile). The samples were collected, dried and then redissolved in 0.1 M TEAB buffer. TMT sixplex labelling reagent was added according to the instructions of manufacturer (Thermo Fisher Scientific Inc., Waltham, MA, USA). After shaking for 2 h, the reaction was stopped with 8% ammonia (Baoruijie, Beijing, China), desalted and lyophilized.

Mobile phases A (2% acetonitrile) and B (98% acetonitrile) were prepared for gradient elution. The sample powder obtained in the previous step was dissolved in mobile phase A and centrifuged at 12,000× *g* for 10 min. The sample was then fractionated by a C18 column (Waters BEH C18, 4.6 × 250 mm, 5 μm, Waters Corp., Milford, MA, USA) on a Rigol L3000 HPLC system (Rigol, Inc., Beijing, China). The elution gradient is shown in Table 1.

### 2.5. LC–MS/MS Analysis

Proteomics analysis was performed using an EASY-nLCTM 1200 UHPLC system (Thermo Fisher). Samples (1 μg) were injected into a C18 Nano-Trap column (4.5 cm × 75 μm, 3 μm) and separated in an analytical column (15 cm × 150 μm, 1.9 μm). Linear gradient elution is shown in Table 2. Following this, the separated peptides were analysed by a Q ExactiveTM HF-X mass spectrometer (Thermo Fisher). The spray voltage was set at 2.1 kV, and the ion transport capillary temperature was 320 °C. The full scan ranged from *m*/*z* 350 to 1500 with a resolution of 60,000 (at *m*/*z* 200). Next, the top 40 abundant precursors were chosen and fragmented by high-energy collisional dissociation (HCD) and analysed by MS/MS (resolution was 3000 (at *m*/*z* 200) for 6 plex). The isolation width was set as 1.0 *m*/*z*.

### 2.6. Bioinformatics Analysis

Raw data files were uploaded via the iProX partner repository [32] with the dataset identifier PXD032898. Proteome Discoverer 2.2 (PD 2.2, Thermo Fisher Scientific) was used to search the resulting spectra from each run. The mass tolerance for the precursor ion was 10 ppm, and that for the product was 0.02 Da. To improve the confidence and quality of the results, peptide spectrum matches (PSMs) with 99% or higher credibility and proteins with at least 1 unique peptide were selected and further analysed with false discovery rate (FDR) of no more than 1%. The quantitation results were statistically analysed with a T test. Between the two groups, proteins (*p* ≤ 0.05 and fold change (FC) ≥ 1.2, or FC ≤ 0.83) were regarded as differentially expressed proteins (DEPs).

These DEPs were subjected to cluster heatmap, volcano map and enrichment analysis. The protein–protein interaction (PPI) was predicted by the STRING-db server (https://cn.string-db.org/, accessed on 2 January 2022) and visualized by Cytoscape v3.9.0. [33,34]. Gene Ontology (GO) functional analysis was performed with the Interproscan program to interpret the possible biological processes [35]. The Kyoto Encyclopaedia of Genes and Genomes (KEGG) database was used to analyse the protein families and pathways [36].

The pathological targets of AD and PD were obtained from DisGeNET database v7.0 (http://www.disgenet.org/, accessed on 16 April 2022) and GeneCards database (https://www.genecards.org/, accessed on 16 April 2022) [37,38].

### 2.7. Caspase 2 Activity Assay and Molecular Docking

Cells were collected after treatment and lysed in cold lysis buffer for 15 min, followed by centrifugation at 16,000× *g* for 15 min at 4 °C. Cell supernatant was obtained and the quantification for protein concentration was determined by Bradford Protein Concentration Determination kit (Beyotime, Shanghai, China). After that, caspase 2 activity was detected by using the Caspase 2 Activity Assay Kit (Beyotime, Shanghai, China) according to the manufacturers’ protocol.

The 3D structures of proteins were obtained from the Protein Data Bank (PDB) database [39,40] (https://www.rcsb.org/, accessed on 16 March 2022). The water and heteroatoms were removed by using Yinfo Cloud Computing Platform (https://cloud.yinfotek.com/, accessed on 16 March 2022). Subsequently, AutoDock Tools 1.5.7 was used to process the receptors and ligand. Relative grid box parameters are represented in Table 3. Molecular docking was carried out by the Lamarckian genetic algorithm of AutoDock 4.2 software with default values (https://autodock.scripps.edu/, accessed on 16 April 2022) [41]. The best conformations with low binding energy were selected and 3D docking diagrams were displayed by PyMOL 2.5.2. (https://pymol.org/2/, accessed on 16 April 2022).

### 2.8. Statistical Analysis

Proteomics analysis was performed in three biological replicates and the mean value was calculated. The protein quantitation results were statistically analysed by *t*-test. *p* values < 0.05 were considered to be statistically significant. The Caspase 2 Activity Assay was analysed by one-way ANOVA and visualized in GraphPad Prism 9.3.1 (GraphPad, San Diego, CA, USA). Data were shown as the mean ± SEM.

## 3. Results

### 3.1. Identification of Proteins

The raw files were directly imported into PD 2.2 for database retrieval and spectrum peptide and protein quantification. The total numbers of identified peptides and proteins are shown in Table 4. A total of 24,183 unique peptides and 4755 proteins were identified.

A series of quality controls related to peptide matching and the identification of proteins were adopted which shown in Supplementary Materials S1. They showed good quality and accuracy of TMT qualification.

Total spectra: total number of secondary spectra. Matched spectrum: effective spectrum amounts. Peptide: the amount of identified peptide. Identified protein: the amount of identified protein. ALL: the quantifiable total protein numbers of all samples.

### 3.2. Proteomic Expression Profile of Stemazole-Treated Human Neural Stem Cells

**Figure 1:**
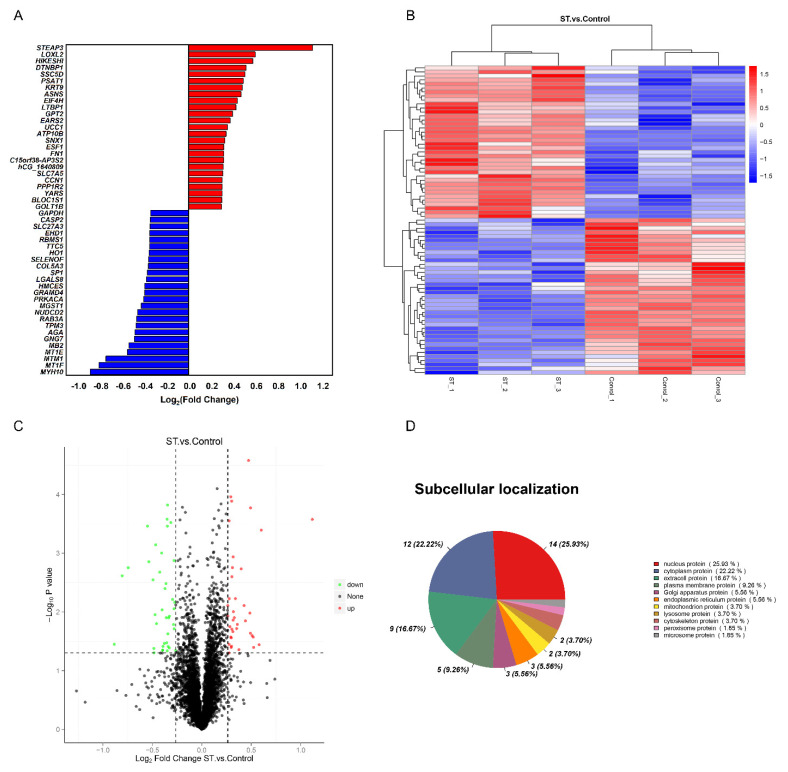
Comparative proteomics analysis of hNSCs in different treatments. (**A**) The top 25 upregulated and downregulated proteins in the stemazole and control groups, satisfying the criteria of fold change (FC) ≥ 1.2 or ≤0.83 and *p* value ≤ 0.05. (**B**) Clustering heatmap of the 77 proteins, in which the expression was altered in different samples. Each row represents one protein, and each column shows one biological replicate. The colour intensity indicates the relative expression level of proteins which by z−value correction. (**C**) Volcano plot distribution of proteins: 38 upregulated (red dots) and 39 downregulated (green dots) proteins in the stemazole−treated cells compared to the control cells. (**D**) Subcellular localization of differentially expressed proteins analysed by the Cell−mPLOC 2.0 program.

To determine the significant difference in the expression of proteins between the two groups, a *t* test was performed, and the *p* value was calculated. The proteins were analysed according to the ratio of mean values of each protein between two groups. When fold change (FC) ≥ 1.2 and *p* ≤ 0.05, the increased proteins were screened, while when FC ≤ 0.83 and *p* ≤ 0.05, the downregulated proteins were screened. Compared to the nutritional deprivation group, 77 DEPs were captured. There were 38 upregulated proteins and 39 downregulated proteins (Table S1). The top 25 differentially expressed proteins (DEPs) compared between the two groups are shown in Figure 1A. A clustering heatmap of proteins whose expression was altered in different samples was constructed. The horizontal axis and vertical axis represent sample and protein clustering, respectively (Figure 1B). The volcano plots provide a global overview of differentially expressed proteins (Figure 1C). Those increased proteins are marked with red dots and decreased with green dots. In addition, the subcellular localization results showed that 25.93% of significantly different proteins were nuclear proteins, 22.22% were cytoplasmic proteins and 16.67% were extracellular proteins (Figure 1D).

### 3.3. Protein–Protein Interaction (PPI) Network Analysis

**Figure 2:**
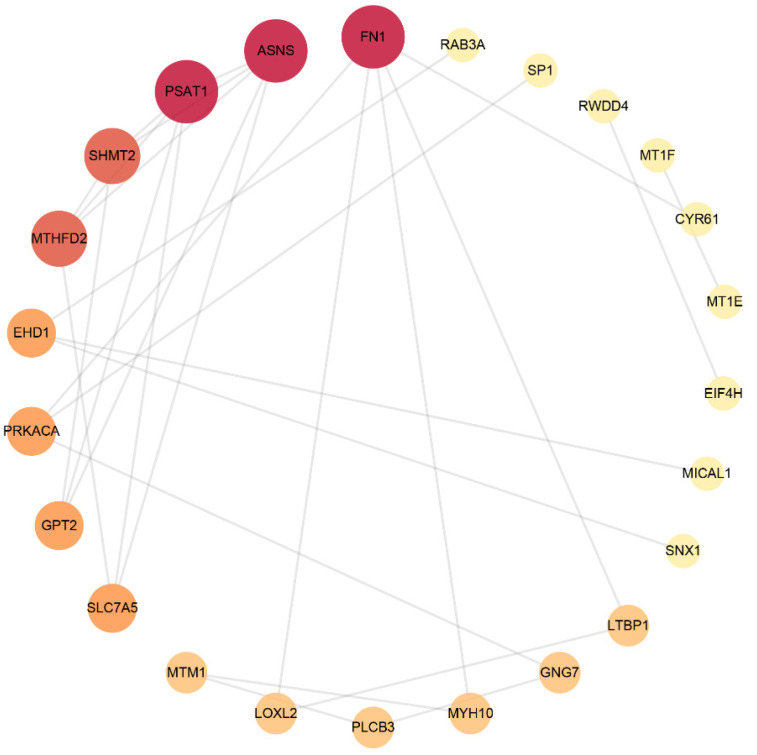
Protein–protein interaction network. The size and colour scales of the circle indicate the node degree centrality. FN1 (fibronectin); PSAT1 (phosphoserine aminotransferase); ASNS (asparagine synthetase); SHMT2 (serine hydroxymethyltransferase); MTHFD2 (bifunctional methylenetetrahydrofolate dehydrogenase/cyclohydrolase, mitochondrial); EHD1 (EH domain-containing protein 1); PRKACA (PKA C-alpha); GPT2 (alanine aminotransferase 2); SLC7A5 (large neutral amino acid transporter small subunit 1); PLCB3 (phospholipase C beta 3).

The STRING database was used to analyse DEPs and interactions among them. There was a score to evaluate the relevance and reliability of protein interactions, and proteins with a confidence score of 0.4 or higher were sent to Cytoscape v3.9.0 to further construct a visual PPI network, which included 56 nodes and 48 edges. As shown in Figure 2 and Table 5, FN1, ASNS, PSAT1, SHMT2, MTHFD2, EHD1, PRKACA, GPT2, SLC7A5 and PLCB3 were the top 10 proteins with a higher network degree value.

### 3.4. Gene Ontology (GO) Functional Annotation and Enrichment Analysis

**Figure 3:**
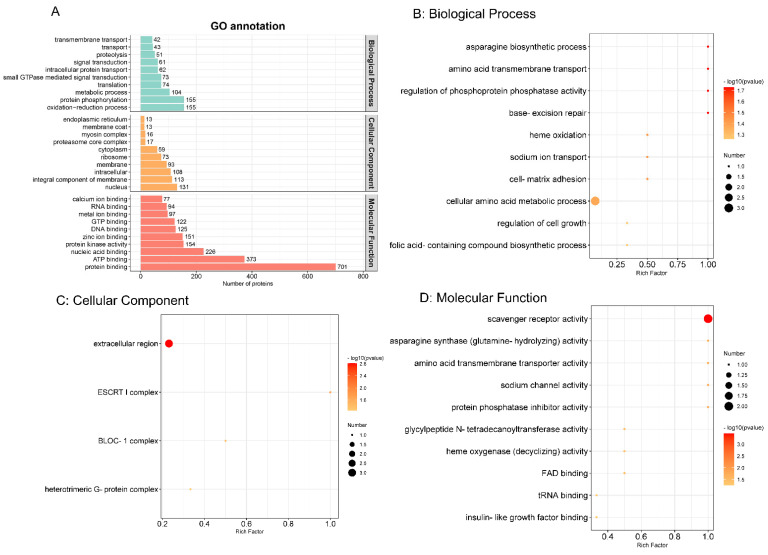
Gene Ontology (GO) functional annotation (**A**) and enrichment analysis (**B**) The top 10 biological processes. (**C**) Four cellular components. (**D**) The top 10 molecular functions. (*p* value ≤ 0.05). The colour scales indicate the different *p* values, and the sizes of the circles represent the number of proteins enriched in each GO term.

The GO functional annotation comprising biological process, cellular component and molecular function were carried out by the Interproscan program. As shown in Figure 3A, in biological process, oxidation–reduction process and protein phosphorylation were the top two annotated GO terms. Nuclear and integral components of the membrane were obvious cellular components. Protein binding and ATP binding were the most enriched molecular functions. To explore the most relevant biological function, GO functional enrichment was conducted with a threshold of *p* ≤ 0.05, as shown in Figure 3B–D. Therefore, in biological process, cellular amino acid metabolic process was the most representative. In the cellular component and molecular function categories, extracellular region and scavenger receptor activity were regarded as the top terms, respectively.

### 3.5. Kyoto Encyclopaedia of Genes and Genomes (KEGG) Pathway Analysis

**Figure 4:**
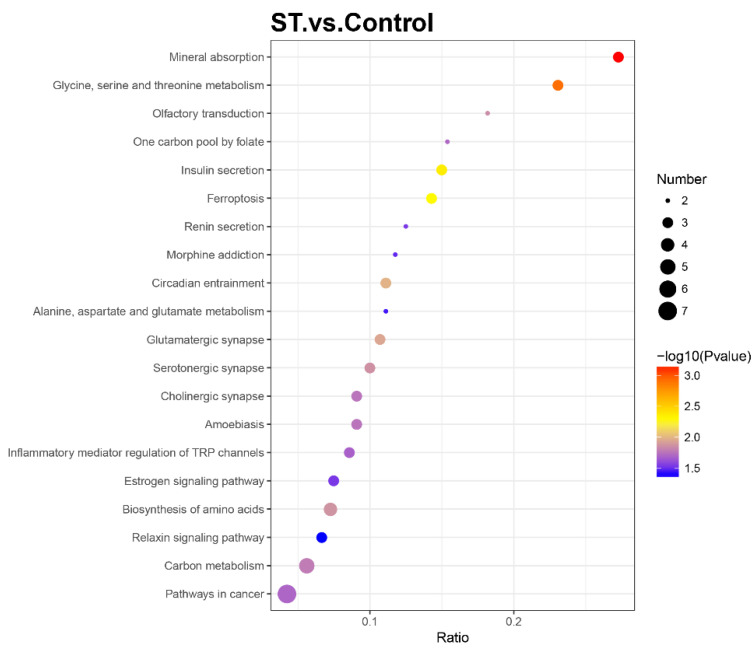
Bubble chart of Kyoto Encyclopaedia of Genes and Genomes (KEGG) enrichment analysis with a *p* value ≤ 0.05. The horizontal axis ratio represents the ratio of differential protein numbers and total numbers identified in this pathway. The colour scales indicate the *p* values of the *t* test, and red represents a higher statistical significance. The sizes of the circles are related to the number of DEPs enriched in each pathway.

To further determine the relevant biochemical metabolic and signal transduction pathways of different abundances of proteins, the screened proteins were matched with annotated proteins in the KEGG pathway database. The upregulated proteins were involved in 32 KEGG pathways, and the downregulated proteins were involved in 121 KEGG pathways. Each pathway was enriched to at least one differential protein (Table S2). Metabolic pathways were primary and involved 13 DEPs, including 6 up- and 7 downregulated differential proteins. The pathways related to neurodegenerative disease, such as Alzheimer’s disease and Huntington’s disease, were also enriched with two essential DEPs (GAPDH, PLCB3 and SP1, PLCB3, respectively). The apoptosis (CASP2) and calcium signalling pathways (PRKACA, PLCB3) also participated as well as the PI3K/AKT/mTOR signalling pathway (FN1, SLC7A5, GNG7). The KEGG enrichment bubble chart was drawn to evaluate the relevance and reliability among proteins and corresponding pathways, which is shown in Figure 4.

### 3.6. Screening of Potential Targets Combined with Diseases Databases

A database of Alzheimer’s disease was constructed to further establish the essential proteins. A total of 3397 and 4799 targets were obtained from DisGeNET database v7.0 and GeneCards database, respectively (Table S3). The intersection between two databases were picked out and regarded as important disease targets. Combined with the omics data, these 77 DEPs made intersection with these 2058 targets and finally six DEPs were found, as shown in Table 6.

### 3.7. Verification of Proteins and Molecular Docking

After making a comprehensive consideration of the PPI network and the anti-apoptotic effects of this molecule in previous work, FN1, PRKACA, SLC7A5 and CASP2 were chosen to be further investigated.

#### 3.7.1. Caspase 2 Activity Assay

**Figure 5:**
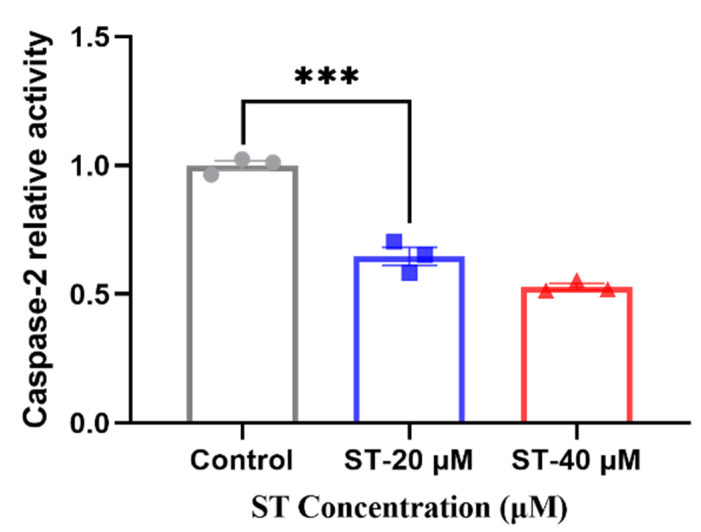
Caspase 2 activity was determined. Data are shown as the mean ± SEM (*n* = 3), *** *p* < 0.001 analysed by one-way ANOVA.

The enzyme activity assay was taken to verify the expression level of CASP2 after adding stemazole. Compared with 40 μM of stemazole in proteomic study, we also added another concentration 20 μM to further investigate the different protein expression level compared to control group. When adding stemazole to reach the target concentration, it can significantly decrease the protein level by 35% and 46%, respectively, which indicated the caspase 2 is a key factor for regulating the survival of human neural stem cells affected by stemazole (Figure 5). 

#### 3.7.2. Molecular Docking

Docking studies were performed between FN1, PRKACA, CASP2, SLC7A5 and stemazole. The results were shown in Table 7, which presented an order from highest to lowest binding energy. The binding modes show that there are hydrophobic interactions and polar interactions between stemazole and these proteins. The molecule could contact them tightly by forming hydrogen bonds and embedding into hydrophobic cavities. 

**Figure 6:**
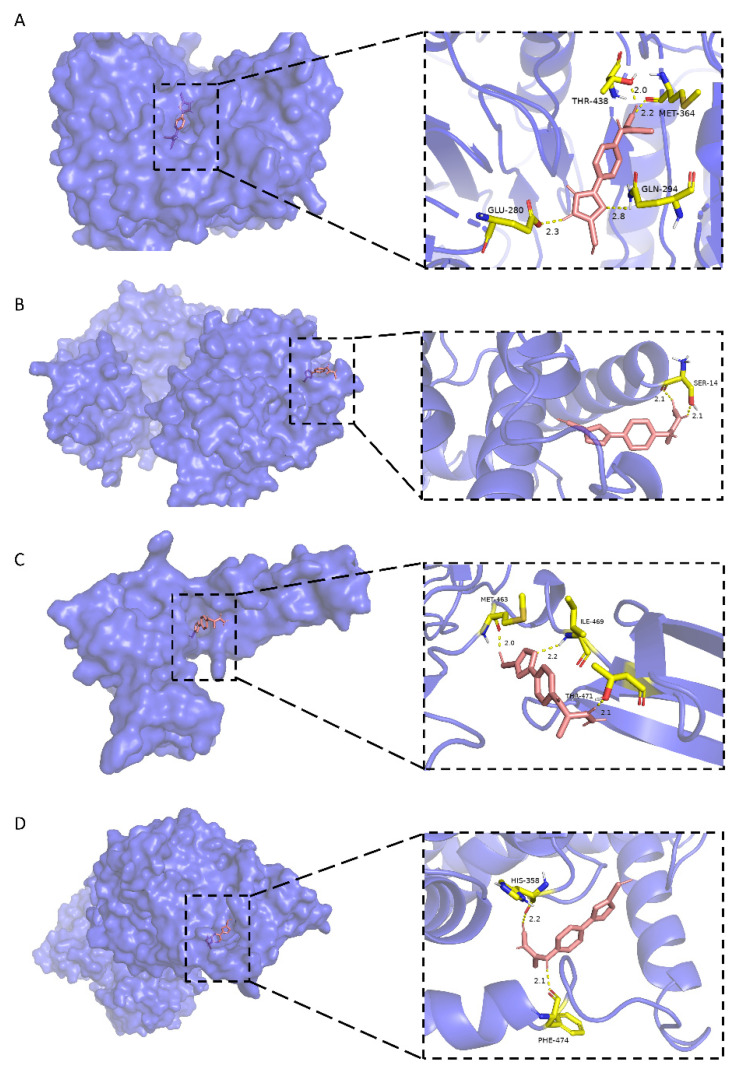
Molecular docking models of stemazole binding to (**A**) CASP2, (**B**) PRKACA, (**C**) FN1 and (**D**) SLC7A5, which are shown as 3D diagrams.

As shown in Figure 6A, stemazole is embedded in a hydrophobic cavity and forms four hydrogen bonds with THR-438 (2.0 Å), MET-364 (2.2 Å), GLN-294 (2.8 Å) and GLU-280 (2.3 Å) of the CASP2 main chains. These interactions stabilize the binding modes and have a low binding energy of −5.46 kcal·moL^−1^.

In Figure 6B, the H1 atom of stemazole contacts the hydroxyl oxygen and carbonyl oxygen of the SER-14 residue of PRKACA to form two hydrogen bonds (2.1 Å and 2.1 Å, respectively). 

Stemazole is bound to a pocket of FN1 through hydrophobic interactions and furthermore stabilized by forming three H-bonds (MET-463, 2.0 Å; THR-471, 2.1 Å; ILE-469, 2.2 Å) which is represented in Figure 6C. 

Moreover, the binding modes of SLC7A5 and stemazole also include hydrophobic interactions and polar interactions. They form two hydrogen bonds which are located in HIS-358 (2.2 Å) and PHE-474 (2.1 Å) residues (Figure 6D).

All these results, which show that stemazole showed good binding affinity to these targets, demonstrate this small molecule may exhibit its role through those receptors that are related to cell proliferation, apoptosis and neurodegenerative diseases. 

## 4. Discussion

With the goal of treating severe neurodegenerative diseases that have no curative efficacy, novel strategies need to be developed. Stem cell therapy seems to be a potential therapeutic measure. Human neural stem cells, which have self-renewal and directed differentiation functions, can replace damaged neural circuits and secrete neurotrophic factors to counter symptomatic deterioration, improve protein levels and reconstitute neural networks. Stemazole is a new small molecule that keeps hNSCs alive in starved cultures and has neuroprotective effects in animal models of Alzheimer’s diseases and Parkinson’s diseases [19,20,21,22,23]. Here, a global protein expression profile of neural stem cells with or without stemazole in conditions of supplement deficiency was conducted to uncover the possible mechanism of stemazole. According to TMT-based proteomics analysis, 77 differentially abundant proteins were found. The 38 upregulated proteins (fold change ≥ 1.2) and 39 downregulated proteins (fold change ≤ 0.83) were involved. Bioinformatics analyses, such as PPI network construction and analysis, GO functional annotation and KEGG pathway annotation and enrichment analysis, were then performed to elucidate the possible biological processes affected by stemazole. The obtained results are authentic and credible, as they came from different databases, information sources and identification methods. Moreover, in order to figure out the key proteins and pathways, we combined our results with the diseases database of AD and screened out four proteins (FN1, PRKACA, CASP2 and SLC7A5). The validations were conducted through Caspase 2 Activity Assay and Molecular Docking. All these results provide a better understanding of the role of stemazole in promoting the survival of hNSCs.

In this study, a PPI network was built to explore the correlation among the DEPs, in which FN1, PSAT1 and ASNS were found to be the key proteins with the highest node degree and centrality, that were all upregulated in hNSCs after treatment with stemazole. Combined with the diseases databases of AD, we obtained four important DEPs, which we investigated by functional analysis (CASP2) and molecular docking (CASP2, PRKACA, FN1, SLC7A5). Stemazole shows low binding energy and good affinity to these targets. The caspase activity assay also validated the reliability and authenticity of proteomics study. 

Fibronectin (FN1) is a member of the glycoprotein family and belongs to the extracellular region of the cellular component in GO annotation. It is regarded as an important component in the PI3K/AKT signalling and cancer pathways [42]. By suppressing the expression of FN1, it can effectively arrest the cell cycle, reduce cell proliferation and migration, and induce cell apoptosis [43,44]. A number of studies have also shown that the extracellular matrix (ECM) microenvironment plays a vital role in regulating the fate of stem cells [45,46]. This protein, as one of the major fibrillary components in ECM, is involved in the proliferation and differentiation of stem cells and is applied in regeneration engineering [47,48]. Blackberry-digested polyphenols (BDPs) extracted from blackberry have been confirmed to promote protective actions in neuronal cells. Inês Figueira et al. [49] conducted transcriptomics to identify major differentially expressed genes and found that proteins in the asparagine and serine pathways, including SLC7A5, ASNS, PSAT1 and SHMT2, were significantly upregulated by BDP treatment. SLC7A5, a crucial amino acid, serves as a transporter for the uptake of large neutral amino acids [50]. The activation of the AKT/mTOR signalling pathway is associated with the transportation of amino acids including leucine by SLC7A5. The overexpression of this protein could elevate the levels of p-AKT and p-mTOR, and thus promote cell proliferation [51,52]. 

Based on this, these overexpressed proteins affected by stemazole may be the core factors regulating the survival of hNSCs.

The two downregulated proteins, PKA C-alpha (PRKACA) and caspase-2 (CASP2), which belong to the protein kinase A (PKA) and caspase families, were further analysed. 

PKA is also known as cAMP-dependent protein kinase A. It is involved in the signal transduction of apoptosis. cAMP induces PKA activation, mediates the apoptosis target genes Bcl-2 and Bax, and promotes cellular apoptosis [53,54]. In this process, the involvement of intracellular calcium seems to be indispensable [55,56]. Various stimuli may trigger calcium overload, further leading to the release of cytochrome C and apoptosis-inducing factors [57]. Evidence indicates that the occurrence of neurodegenerative disease is also related to intracellular calcium dyshomeostasis [58,59,60]. PKA plays a key role in the Parkinson’s disease signalling pathway, which is associated with striatal output activity and synaptic plasticity [61]. It has been confirmed that the increase in cAMP/PKA in the striatum is highly relevant to the loss of dopaminergic neurons in PD rats [62].

Therefore, the low expression levels of PRKACA caused by this molecule may alleviate cell calcium dyshomeostasis and apoptosis in the absence of growth factors.

The caspase family is well known to be involved in apoptosis (programmed cell death), necrosis and inflammation [63,64,65]. Caspase 2 protein was significantly downregulated in the omics data. It acts upstream of mitochondria, promotes the release of cytochrome c and induces apoptosis before the change in mitochondrial permeability [66,67]. Studies have also shown that the activation of caspase 2 occurs before caspase 3, and caspase 2 is cleaved by active caspase 3. The precise mechanism by which the caspase 2 pathway leads to apoptosis remains controversial [68,69]. However, it is still worth noting that caspase 2 mediates the neural cell death initiated by β-amyloid cytotoxicity and acts as both the initiator and effector to apoptosis, which is different from caspase 3 and the canonical cascade pathway [70]. The protein levels of caspase 2 are significantly upregulated in AD brains, and it is a critical driver in synaptic dysfunction by activating the RhoA/ROCK-II signalling pathway, whereas reducing caspase 2 levels restores memory function [71,72,73]. These findings all indicate that caspase 2 offers novel therapeutic targets in the treatment of neurological disorders. 

In this work, caspase 2 had a low expression level in the stemazole-treated group. The validation experiment of this protein also confirmed that the addition of stemazole in the absence of growth factors significantly decreased the expression of CASP2. All these studies demonstrated CASP2 may be a key factor in stemazole’s anti-apoptosis effects and ability to treat neurodegenerative diseases.

To sum up, they may serve as regulators in the treatment of neurodegenerative disorders and provide new ideas for the treatment of neurodegenerative diseases from stem cell drugs.

GO and KEGG enrichment analysis provided further knowledge of biological processes and important pathways that could lead to a deep understanding of the way this molecule works.

Cells must have macromolecular precursors such as amino acids to sustain growth, whereas in amino acid deprivation or nutrient stress, the biosynthesis of amino acid processes and metabolic processes are obviously affected [74]. DEPs enriched in these pathways also control the fate of cell proliferation and apoptosis. Meanwhile, some overexpressed or suppressed proteins which regulate the fate of tumour cells have gained more attention in cancer research; thus, they are often enriched in cancer pathways. However, from another perspective, FN1, PRKACA were enriched in cancer pathways in this work, indicating that stemazole may affect the growth of hNSCs, instead of tumour cells, with a positive side. 

There were two different outcomes in the stemazole treatments ((1) the other conditions were not changed; (2) growth factors were removed). Stemazole did not exhibit superimposed proliferative effects under common conditions and even showed cytotoxicity at high concentrations, but in the absence of growth factors, the neuroprotective effect of stemazole was particularly obvious. These results also remind us that the role was masked in nutrient-sufficient conditions and that stemazole exhibits a major role in rescuing cells from apoptosis under disaggregation or starvation. This should be a focus in future research.

Briefly, this study was performed to continue to explore the anti-apoptotic effects of stemazole on hNSCs in the absence of supplements. The addition of stemazole revealed a series of changes in biological metabolic processes as well as some crucial proteins involved in cell proliferation, apoptosis and some neurodegenerative diseases. It is possible that this small molecule plays roles by multiple interactions and pathways. Key proteins and related pathways will need to be verified by other techniques in the future.

## 5. Conclusions

The underlying mechanisms of stemazole in its human neural stem cell protective effects were investigated by conducting proteomics and bioinformatics analyses. The addition of stemazole altered the protein expression profiles of hNSCs under extreme conditions. A total of 77 differentially expressed proteins were screened, including 38 upregulated proteins and 39 downregulated proteins. The key proteins CASP2, PRKACA, FN1 and SLC7A5 were further identified and validated. In summary, this is the first systematic study of the whole protein expression profiles of hNSCs in the absence of growth factors and presence of stemazole. The findings might present a basis and were aimed at deepening the anti-apoptotic and neuroprotective profile of stemazole. They also provide a foundation and guidelines for the exploration of the mechanism in an animal disease model as well as new insights into the current treatment of neurodegenerative disorders using stem cell drugs. Key biological processes and pathways should be examined in future research.

## Figures and Tables

**Table 1:** Liquid chromatography elution gradient table of peptide fraction separation.

Time (min)	Flow Rate (mL/min)	Mobile Phase A (%)	Mobile Phase B (%)
0	1	97	3
10	1	95	5
30	1	80	20
48	1	60	40
50	1	50	50
53	1	30	70
54	1	0	100

**Table 2:** Liquid chromatography elution gradient table.

Time (min)	Flow Rate (nL/min)	Mobile Phase A (%)	Mobile Phase B (%)
0	600	94	6
2	600	85	15
48	600	60	40
50	600	50	50
51	600	45	55
60	600	0	100

**Table 3:** Parameters of the grid box.

Receptors	PDB ID		Centre Grid Box	
CASP2	3R5J	−8.092	−3.584	23.246
PRKACA	5IZJ	23.518	4.957	98.333
FN1	3MQL	−12.569	−17.608	34.877
SLC7A5	7DSK	139.405	143.039	157.114

**Table 4:** An overview of protein identification.

	Total Spectra	Matched Spectrum	Peptide	Identified Protein	ALL
Run 1	294,458	33,410	24,183	4755	4747

**Table 5:** The relative parameters of the protein–protein interaction network.

	Degree	Betweenness Centrality	Closeness Centrality	Neighbourhood Connectivity
FN1	5	0.6528	0.6429	2.0000
PSAT1	5	0.1167	1.0000	3.8000
ASNS	5	0.1167	1.0000	3.8000
SHMT2	4	0.0333	0.8333	4.2500
MTHFD2	4	0.0333	0.8333	4.2500
EHD1	3	1.0000	1.0000	1.0000
PRKACA	3	0.4028	0.5625	2.6667
GPT2	3	0.0000	0.7143	4.6667
SLC7A5	3	0.0000	0.7143	4.6667
PLCB3	2	0.0694	0.3750	2.0000
MYH10	2	0.1944	0.5000	3.5000
LTBP1	2	0.0000	0.4286	3.5000
MTM1	2	0.0972	0.4091	2.0000
GNG7	2	0.1389	0.4500	2.5000
LOXL2	2	0.0000	0.4286	3.5000
SNX1	1	0.0000	0.6000	3.0000
CYR61	1	0.0000	0.4091	5.0000
RWDD4	1	0.0000	1.0000	1.0000
MT1F	1	0.0000	1.0000	1.0000
MICAL1	1	0.0000	0.6000	3.0000
MT1E	1	0.0000	1.0000	1.0000
RAB3A	1	0.0000	0.6000	3.0000
SP1	1	0.0000	0.3750	3.0000
EIF4H	1	0.0000	1.0000	1.0000

**Table 6:** The six potential targets of stemazole combined with diseases databases.

No.	Symbol	Protein Name
1	CASP2	Caspase-2
2	PRKACA	PKA C-alpha
3	FN1	Fibronectin
4	SLC7A5	large neutral amino acid transporter small subunit 1
5	RAB3A	Ras-related protein Rab-3A
6	SP1	Transcription factor sp1

**Table 7:** The results of molecular docking between receptors and ligand.

Receptors	Binding Energy (∆G)/kcal·moL^−1^	RMSD (Å)
FN1	−5.90	1.489
PRKACA	−5.75	0.622
CASP2	−5.46	1.000
SLC7A5	−4.31	1.275

## Data Availability

The mass spectrometry proteomics data have been deposited with the ProteomeXchange Consortium (http://proteomecentral.proteomexchange.org, accessed on 3 February 2022) via the iProX partner repository [32] with the dataset identifier PXD032898.

## Supplementary Materials

The following supporting information can be downloaded at https://www.mdpi.com/article/10.3390/brainsci12060690/s1, Supplementary Materials S1: Quality control of protein identification; Figure S1: Human neural stem cell viability affected by stemazole at a series of concentrations in the absence of growth factors and quantified by CellTiter-Glo^®^; Table S1: Summary results for the differential expression of proteins; Table S2: KEGG pathways involved in the differential expression of proteins. Each pathway involved at least one protein; Table S3: Targets associated with Alzheimer’s disease from different databases.

## Abbreviations

AD	Alzheimer’s disease
ASNS	asparagine synthetase
bFGF	basic fibroblast growth factor
CASP2	caspase-2
CASP3	caspase-3
CASP8	caspase-8
CASP9	caspase-9
DAG	diacylglycerol
DEPs	differentially expressed proteins
EGF	epidermal growth factor
EHD1	EH domain-containing protein 1
FC	fold change
FDR	false discovery rate
FN1	fibronectin
GAPDH	glyceraldehyde-3-phosphate dehydrogenase
GNG7	guanine nucleotide-binding protein G(I)/G(S)/G(O) subunit gamma-7
GO	gene ontology
GPT2	alanine aminotransferase 2
hNSCs	human neural stem cells
IP3	inositol 1,4,5-triphosphate
KEGG	Kyoto Encyclopaedia of Genes and Genomes
MTHFD2	bifunctional methylenetetrahydrofolate dehydrogenase/cyclohydrolase, mitochondrial
MYH10	myosin-10
PD	Parkinson’s disease
PKA	protein kinase A
PKC	protein kinase C
PLC	phospholipase C
PLCB3	phospholipase C beta3
PPI	protein–protein interaction
PRKACA	PKA C-alpha
PSAT1	phosphoserine aminotransferase
SHMT2	serine hydroxymethyltransferase
SLC7A5	large neutral amino acid transporter small subunit 1
SP1	transcription factor Sp1
ST	stemazole
TMT	tandem mass tags
