# Supplementary material for: A Quantitative Proteomic Approach Explores the Possible Mechanisms by Which the Small Molecule Stemazole Promotes the Survival of Human Neural Stem Cells
Source: Brain Sci. 2022 May 25;12(6):690. doi: 10.3390/brainsci12060690 (PMC9221083; doi:10.3390/brainsci12060690)
Supplement: Supplementary file 1 [file brainsci-12-00690-s001.zip › brainsci-1712829-supplementary.pdf]

Supplementary material S1----Quality control of protein identification

The Peptides Length Distribution reflected the selection of an appropriate enzyme. As shown here in Figure SA (Supplementary Material 1), the length of the peptides mainly ranged from 7 to 25, which can effectively be detected by mass spectrometer.

In MS data acquisition, precursor ion tolerance distribution was evaluated to be an indicator of MS performance and a reference for the quality of the identified results. There is deviation between the precursor ion detected by MS and theoretical molecular weight. The peak being near zero indicates a subtle mass deviation (Figure SB).

The identified peptides and proteins were matched with a protein database. Proteins containing exactly the same peptides were called the same group proteins, and have unique peptides in each group. The more unique peptides, the more reliable the identified proteins. In Figure SC, the X-axis represents the number of unique peptides, while the Y-axis shows the ratio of proteins containing unique peptides and total proteins as the increase of the number of unique peptides. Thus, the more slowly the curve increases, the more unique peptides and more reliable proteins are identified.

With regard to an identified protein, there are as many unique peptides as possible which indicates the reliability of the protein. Figure SD represents the protein coverage and the horizontal axis means the length of detecting peptides/the whole length of this protein. Protein mass is also an important indicator and the wider the molecular weight range, the wider the range of proteins identified, as shown in Figure SE.

Overall, we have taken measures to ensure the reliability and accuracy of the data from sample preparation and TMT labelling to data analysis.

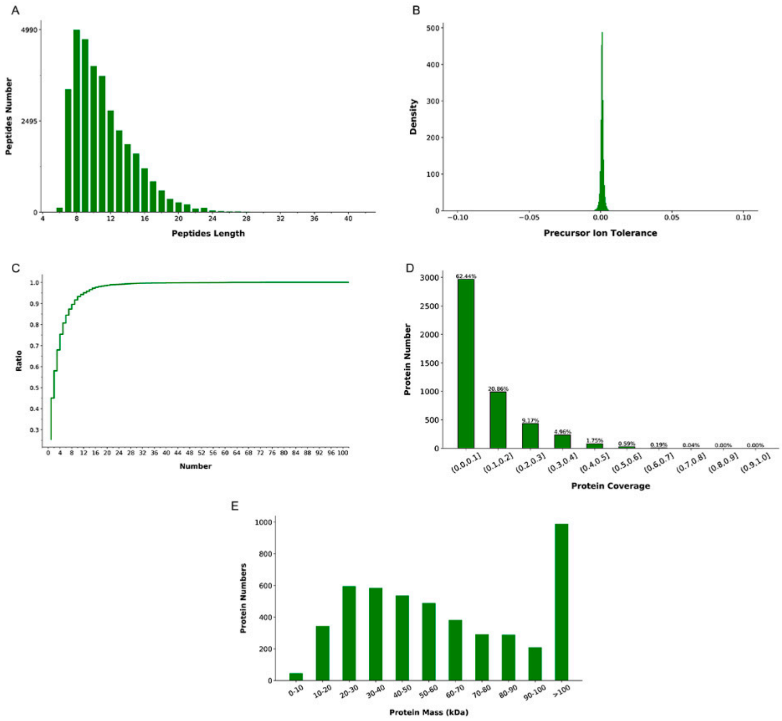

Figure S: Quality control of protein identification. (A) Peptide length range distribution. (B) Precursor ion tolerance plot. (C) The distribution of unique peptide numbers. (D) The coverage of identified proteins. (E) Protein molecular weight distribution.

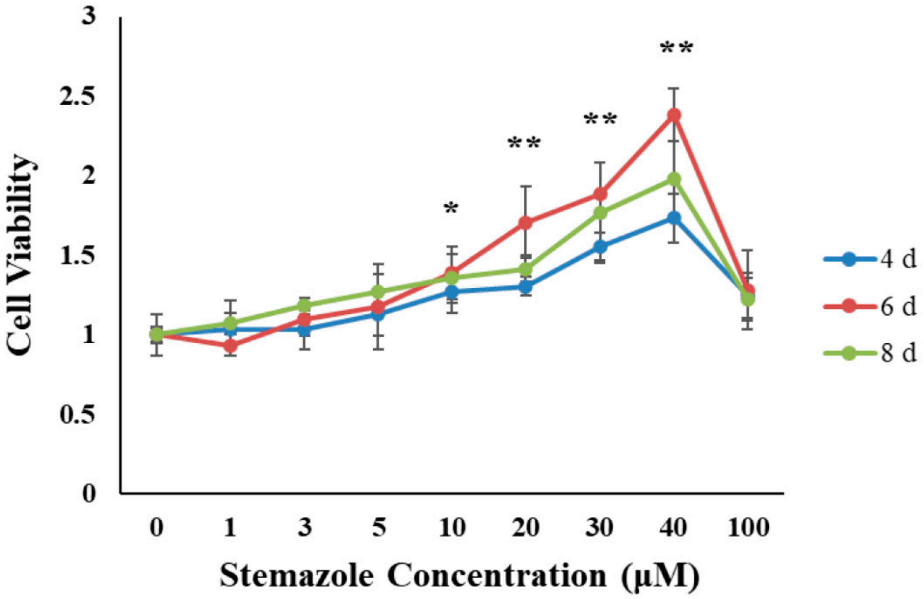

Figure S1. Human neural stem cell viability affected by stemazole at a series of concentrations in the absence of growth factors and quantified by CellTiter-Glo®.

Table S1. Summary results for the differential expression of proteins.

Protein	Description	Gene	ST_1	ST_2	ST_3	Conrol_1	Conrol_2	Conrol_3	ST.vs.Control FC	ST.vs.Control P-value	ST.vs.Control log2FC	ST.vs.Control UP.DOWN
B3KUB6	cDNA FLJ39529 fis, clone PUAEN2004067, highly similar to Band 4.1-like protein 1		148.9	135.9	144	126.8	101.8	125.5	1.210957	0.049963	0.276148	up
A0A0S2Z4R1	Tyrosine--tRNA ligase (Fragment)	YARS	1801.5	1726	1762.2	1423.9	1444.2	1422.8	1.232772	0.00013	0.301906	up
P35580	Myosin-10	MYH10	48.7	49.7	48.2	103	92.5	75.3	0.541359	0.03565	-0.88534	down
A0A0S2Z4I4	Tropomyosin 3 isoform 3 (Fragment)	TPM3	8.4	9.6	10.4	12.2	13	14.2	0.720812	0.011151	-0.4723	down
Q13596	Sorting nexin-1	SNX1	1106.6	1028.5	1097.5	887.7	769.9	915.5	1.256306	0.01253	0.329187	up
P08243	Asparagine synthetase [glutamine-hydrolyzing]	ASNS	844.9	873.3	875.7	613	629.1	626.9	1.387854	2.64E-05	0.472856	up
P34897	Serine hydroxymethyltransferase, mitochondrial	SHMT2	1255.1	1304.4	1263	1031.5	1062.6	1063.3	1.210648	0.00028	0.275779	up
O95036	Similar to 60S ribosomal protein L7 similar to P18124 (PID:d133021)	WUGSC:H_RG054D04.1	64.4	67.5	57.4	50.3	49.5	54.7	1.225243	0.026898	0.293068	up
P02751	Fibronectin	FN1	593.9	587	631.7	491.8	478.8	483.8	1.246287	0.001155	0.317636	up
A0A024RBB7	Nucleosome assembly protein 1-like 1, isoform CRA_a	NAP1L1	765.3	755.6	745.5	628	618.4	603.5	1.225147	0.000109	0.292955	up
B4DHQ3	Phosphoserine aminotransferase		456.7	442.8	466.3	337.1	316.9	315.9	1.408186	0.000169	0.493838	up
P20336	Ras-related protein Rab-3A	RAB3A	76.2	68.6	68.3	96.7	100.7	97	0.723845	0.000718	-0.46625	down
P35527	Keratin, type I cytoskeletal 9	KRT9	269.1	253.1	267	156.1	204	203.1	1.401278	0.010504	0.486744	up
P30419	Glycylpeptide N-tetradecanoyltransferase 1	NMT1	58.6	58.7	62.4	51.8	46.1	51.8	1.200401	0.011722	0.263516	up
A0A0A6YYH1	Protein C15orf38-AP3S2	C15orf38-AP3S2	283.8	303.1	290.1	252	210.2	241.5	1.24627	0.013786	0.317616	up
A0A024R571	EH domain-containing protein 1	EHD1	236.7	228.3	224.4	296.3	284.1	296.9	0.78582	0.000346	-0.34773	down
A0A024R7J0	Protein kinase, cAMP-dependent, catalytic, alpha, isoform CRA_c	PRKACA	19.2	18.9	17.9	25.4	22.3	26.3	0.756757	0.009234	-0.4021	down
X6R3N0	Solute carrier family 27 (Fatty acid transporter), member 3, isoform CRA_d	SLC27A3	253.6	248.6	251.2	328	312	318.2	0.786266	0.000151	-0.34691	down

A0A3F2YNW7	AT-rich interactive domain-containing protein 1B	ARID1B	294.8	298.5	273.7	355.2	331.9	361.1	0.827132	0.006883	-0.27381	down
B2RBE0	cDNA, FLJ95462, highly similar to Homo sapiens fatty-acid-Coenzyme A ligase, long-chain 3 (FACL3),mRNA		107.8	121.1	111.9	150.1	127.3	132.1	0.832234	0.045425	-0.26494	down
P04733	Metallothionein-1F	MT1F	91.3	91.5	91.9	166.2	159.5	154.4	0.572172	0.002426	-0.80548	down
Q59G91	TC10-like Rho GTPase variant (Fragment)		31.4	32.6	34.1	36.5	44.2	48.5	0.759288	0.044885	-0.39728	down
Q2TSD0	Glyceraldehyde-3-phosphate dehydrogenase		35.7	37.4	43.3	45	53.7	48.7	0.789688	0.038916	-0.34065	down
H0YHG0	Uncharacterized protein (Fragment)		190.3	157.3	156.4	133.9	107.7	116.8	1.40625	0.023084	0.491853	up
Q01970	1-phosphatidylinositol 4,5-bisphosphate phosphodiesterase beta-3	PLCB3	410.2	430.9	432.2	522.5	537.1	521.5	0.805325	0.0003	-0.31236	down
P04732	Metallothionein-1E	MT1E	255.3	232	246.1	348.5	352.2	372.1	0.683632	0.000345	-0.54871	down
A0A024R6R2	Glutamic pyruvate transaminase (Alanine aminotransferase) 2, isoform CRA_b	GPT2	113.5	123.7	121.8	86	93.6	92.7	1.318399	0.001846	0.398787	up
E7BJU4	Heme oxygenase 1 (Fragment)	HO1	70.9	67.2	85.5	96.6	88.9	100.4	0.782092	0.033541	-0.35459	down
P84101	Small EDRK-rich factor 2	SERF2	127.2	114	124.8	157.7	148.2	138.4	0.823768	0.019335	-0.27969	down
Q8TDZ2	[F-actin]-monooxygenase MICAL1	MICAL1	125.3	129.3	134.2	168.7	156.2	152.3	0.814753	0.006151	-0.29557	down
P78537	Biogenesis of lysosome-related organelles complex 1 subunit 1	BLOC1S1	15.3	14.4	17.6	12.9	12.4	13.1	1.231771	0.038329	0.300734	up
Q9HC85	Metastasis related protein (Fragment)	MB2	134.4	146.7	130.4	186.7	204.3	205.6	0.689742	0.001398	-0.53587	down
A1KY36	Cell proliferation-inducing protein 41		115.8	95.8	107.2	81.7	82	84.8	1.282897	0.016291	0.359406	up
A8K139	cDNA FLJ76744, highly similar to Homo sapiens L1 cell adhesion molecule (L1CAM), transcript variant 1, mRNA		117.9	98.2	102.6	55.9	82.8	83	1.437528	0.040188	0.52359	up
Q8N0Z6	Tetratricopeptide repeat protein 5	TTC5	47.1	48	49.9	61.6	62.9	60.4	0.784208	0.000265	-0.35069	down

H0YI09	Methyltransferase-like protein 7A (Fragment)	METTL7A	42.6	40.4	36.1	46.5	47.6	55.5	0.796123	0.040938	-0.32894	down
Q8TE01	DERP12 (Dermal papilla derived protein 12)	derp12	117.4	107.7	114.7	137.7	135.9	139.3	0.82296	0.001335	-0.28111	down
F8W0Q9	Periphilin-1	PPHLN1	87.3	98.9	94.8	116.6	108.8	116.1	0.82284	0.008853	-0.28132	down
Q15056	Eukaryotic translation initiation factor 4H	EIF4H	101	95.3	103.8	83.6	63.4	74	1.357919	0.014213	0.441397	up
Q01650	Large neutral amino acids transporter small subunit 1	SLC7A5	174.2	169.2	167.2	143.8	130.3	137.9	1.23932	0.001758	0.309549	up
Q9HA47	Uridine-cytidine kinase 1	UCK1	95.5	99.2	115.3	127.2	132.3	131.7	0.792434	0.012596	-0.33564	down
Q14766	Latent-transforming growth factor beta-binding protein 1	LTBP1	60.8	67	61.1	47.6	42	50.8	1.345442	0.007788	0.42808	up
A8K1U5	cDNA FLJ75602, highly similar to Homo sapiens amiloride-sensitive cation channel 2, neuronal (ACCN2), transcript variant 2, mRNA		87.9	95	93.4	122.5	126.5	117.1	0.754712	0.000993	-0.406	down
A4D1W8	Ependymin related protein 1 (Zebrafish), isoform CRA_b	UCC1	89.5	75.4	78.6	66.8	63.4	60.5	1.276875	0.019216	0.352617	up
Q658P3	Metalloreductase STEAP3	STEAP3	66.8	62.9	57.6	27.4	28.8	30.1	2.170336	0.000266	1.117918	up
O60613	Selenoprotein F	SELENOF	29.2	31.8	29.5	35.1	40.4	40.6	0.7795	0.012534	-0.35938	down
Q53FT3	Protein Hikeshi	HIKESHI	151.1	124	111.6	71.7	91.7	95.2	1.49536	0.036186	0.580492	up
P42575	Caspase-2	CASP2	89.2	111.6	107.3	128.8	137.1	124.9	0.788383	0.023638	-0.34303	down
P41236	Protein phosphatase inhibitor 2	PPP1R2	64.7	58.3	62.7	46.8	55	48.6	1.234707	0.01968	0.304169	up
A0A024RD41	RAB23, member RAS oncogene family, isoform CRA_a	RAB23	127.9	120.7	128.3	164	164.9	143.9	0.797166	0.011807	-0.32705	down
A0A024R4N0	HCG1640809, isoform CRA_b	hCG_1640809	40.3	45	38	32.8	31.9	34.5	1.242944	0.02163	0.313761	up
O00622	CCN family member 1	CCN1	88.1	88.4	91.6	76.7	69.1	71.3	1.234915	0.002518	0.304411	up
P13995	Bifunctional methylenetetrahydrofolate dehydrogenase/cyclohydrolase, mitochondrial	MTHFD2	153.5	161.1	177.5	146.2	134.1	126.2	1.210578	0.035743	0.275696	up
B4E2I9	cDNA FLJ55486, highly similar to Semaphorin-3C		78	90.4	83	66.6	71.4	68.4	1.218023	0.017817	0.284542	up

Q9H501	ESF1 homolog	ESF1	204.1	185.1	180.9	147	149.8	160.1	1.247757	0.009905	0.319337	up
P08047	Transcription factor Sp1	SP1	64.7	63.4	62.9	76.4	86.8	83.9	0.772966	0.004012	-0.37152	down
P78549	Endonuclease III-like protein 1	NTHL1	75.1	71.7	63.7	85.6	88.9	82.3	0.819704	0.016421	-0.28682	down
A0A0S2Z5U8	Dystrobrevin binding protein 1 isoform 1 (Fragment)	DTNBP1	10	8.5	8.9	7.4	6.6	5.1	1.434555	0.026853	0.520603	up
A0A024RC06	Myotubularin 1, isoform CRA_a	MTM1	146.9	116.4	131.4	205	232.6	223.8	0.596764	0.001766	-0.74477	down
Q8WVJ2	NudC domain-containing protein 2	NUDCD2	33	33.6	38.5	51.6	50.1	42.7	0.727839	0.015822	-0.45831	down
Q5JPH6	Probable glutamate--tRNA ligase, mitochondrial	EARS2	35.8	31.2	28.7	22.1	27	24.7	1.296748	0.044018	0.374898	up
P20933	N(4)-(beta-N-acetylglucosaminyl)-L-asparaginase	AGA	75.6	93.7	90.9	140.7	120.5	102.5	0.715425	0.049528	-0.48313	down
A0A024R3X1	Galectin	LGALS8	6.2	6.7	5.4	8.6	8.1	7.1	0.768908	0.034366	-0.37912	down
A0A024RAX2	Microsomal glutathione S-transferase 1, isoform CRA_a	MGST1	25.8	24.3	22.2	33.2	31.4	32.5	0.744593	0.002104	-0.42548	down
Q6NW29	RWD domain-containing protein 4	RWDD4	48.9	44.5	48	42.2	33.4	39.5	1.228497	0.040192	0.296894	up
A0A0S2Z4B3	RNA binding motif single stranded interacting protein 1 isoform 2 (Fragment)	RBMS1	7.6	7.9	6.4	9.1	8.5	10.3	0.784946	0.046062	-0.34933	down
P25940	Collagen alpha-3(V) chain	COL5A3	175.2	180	186.3	217.1	237.5	241.5	0.777905	0.00329	-0.36233	down
P28356	Homeobox protein Hox-D9	HOXD9	33.1	32.6	32	26.8	29.1	25.5	1.200246	0.007808	0.26333	up
Q6IC98	GRAM domain-containing protein 4	GRAMD4	14.4	14.6	14.1	20.8	18.6	17.3	0.760141	0.044733	-0.39566	down
Q9Y4K0	Lysyl oxidase homolog 2	LOXL2	91.8	97.8	92	60.7	66.1	58.8	1.517241	0.000405	0.601451	up
A0A024R8B8	Chromosome 9 open reading frame 28, isoform CRA_a	C9orf28	114.2	124.3	116.6	99.2	92.9	100.8	1.212359	0.00594	0.277817	up
Q96FZ2	Abasic site processing protein HMCES	HMCES	26.9	25.4	25.4	30.8	32	39.2	0.761765	0.038649	-0.39258	down
B4DSR7	cDNA FLJ58470, highly similar to SS18-like protein 1		17.2	18.2	20.6	27.2	25.2	26.4	0.71066	0.002845	-0.49277	down
O60262	Guanine nucleotide-binding protein G(I)/G(S)/G(O) subunit gamma-7	GNG7	3.5	4.5	4	4.7	5.9	6.2	0.714286	0.041795	-0.48543	down

O94823	Probable phospholipid-transporting ATPase VB	ATP10B	63.5	65.7	70.3	55.9	50.9	50.8	1.265863	0.005936	0.340121	up
A1L4H1	Soluble scavenger receptor cysteine-rich domain-containing protein SSC5D	SSC5D	9.9	11.3	10.4	9	6.7	6.5	1.423423	0.025373	0.509365	up
Q9Y3E0	Vesicle transport protein GOT1B	GOLT1B	27.6	27.3	23.3	21.1	20.6	21.9	1.22956	0.027603	0.298142	up

Table S2. KEGG pathways involved in the differential expression of proteins. Each pathway involved at least one protein;.

MapID	MapTitle	Pvalue	AdjustedPv	x	y	n	N	EnrichDirect	ProtID	Description
map01100	Metabolic pathways	0.185346	0.448843	13	525	40	2326	Over	P08243 P34897 B4DHQ3 B2RBE0 Q2TSD0 Q01970 A0A024R6R2 E7BJU4 Q8TE01 Q9HA47 P13995 A0A024RC06 Q5JPH6	Asparagine synthetase [glutamine-hydrolyzing],Serine hydroxymethyltransferase, mitochondrial,Phosphoserine aminotransferase,cDNA, FLJ95462, highly similar to Homo sapiens fatty-acid-Coenzyme A ligase, long-chain 3 (FACL3),mRNA,Glyceraldehyde-3-phosphate dehydrogenase,1-phosphatidylinositol 4,5-bisphosphate phosphodiesterase beta-3,Glutamic pyruvate transaminase (Alanine aminotransferase) 2, isoform CRA_b,Heme oxygenase 1 (Fragment),DERP12 (Dermal papilla derived protein 12),Uridine-cytidine kinase 1,Bifunctional methylenetetrahydrofolate dehydrogenase/cyclohydrolase, mitochondrial,Myotubularin 1, isoform CRA_a,Probable glutamate--tRNA ligase, mitochondrial
map05200	Pathways in cancer	0.020176	0.200943	7	165	40	2326	Over	P02751 A0A024R7J0 Q01970 E7BJU4 P08047 A0A024RAX2 O60262	Fibronectin,Protein kinase, cAMP-dependent, catalytic, alpha, isoform CRA_c,1-phosphatidylinositol 4,5-bisphosphate phosphodiesterase beta-3,Heme oxygenase 1 (Fragment),Transcription factor Sp1,Microsomal glutathione S-transferase 1, isoform CRA_a,Guanine nucleotide-binding protein G(I)/G(S)/G(O) subunit gamma-7
map01200	Carbon metabolism	0.016738	0.200943	5	89	40	2326	Over	P34897 B4DHQ3 Q2TSD0 A0A024R6R2 Q8TE01	Serine hydroxymethyltransferase, mitochondrial,Phosphoserine aminotransferase,Glyceraldehyde-3-phosphate dehydrogenase,Glutamic pyruvate transaminase (Alanine aminotransferase) 2, isoform CRA_b,DERP12 (Dermal papilla derived protein 12)
map01230	Biosynthesis of amino acids	0.013601	0.200943	4	55	40	2326	Over	P34897 B4DHQ3 Q2TSD0 A0A024R6R2	Serine hydroxymethyltransferase, mitochondrial,Phosphoserine aminotransferase,Glyceraldehyde-3-phosphate dehydrogenase,Glutamic pyruvate transaminase (Alanine aminotransferase) 2, isoform CRA_b

map04978	Mineral absorption	0.000707	0.085552	3	11	40	2326	Over	P04733 P04732 E7BJU4	Metallothionein-1F,Metallothionein-1E,Heme oxygenase 1 (Fragment)
map00260	Glycine, serine and threonine metabolism	0.001197	0.085552	3	13	40	2326	Over	P34897 B4DHQ3 Q8TE01	Serine hydroxymethyltransferase, mitochondrial,Phosphoserine aminotransferase,DERP12 (Dermal papilla derived protein 12)
map04911	Insulin secretion	0.004387	0.180804	3	20	40	2326	Over	P20336 A0A024R7J0 Q01970	Ras-related protein Rab-3A,Protein kinase, cAMP-dependent, catalytic, alpha, isoform CRA_c,1-phosphatidylinositol 4,5-bisphosphate phosphodiesterase beta-3
map04216	Ferroptosis	0.005057	0.180804	3	21	40	2326	Over	B2RBE0 E7BJU4 Q658P3	cDNA, FLJ95462, highly similar to Homo sapiens fatty-acid-Coenzyme A ligase, long-chain 3 (FACL3),mRNA,Heme oxygenase 1 (Fragment),Metalloreductase STEAP3
map04713	Circadian entrainment	0.010356	0.200943	3	27	40	2326	Over	A0A024R7J0 Q01970 O60262	Protein kinase, cAMP-dependent, catalytic, alpha, isoform CRA_c,1-phosphatidylinositol 4,5-bisphosphate phosphodiesterase beta-3,Guanine nucleotide-binding protein G(I)/G(S)/G(O) subunit gamma-7
map04724	Glutamatergic synapse	0.011462	0.200943	3	28	40	2326	Over	A0A024R7J0 Q01970 O60262	Protein kinase, cAMP-dependent, catalytic, alpha, isoform CRA_c,1-phosphatidylinositol 4,5-bisphosphate phosphodiesterase beta-3,Guanine nucleotide-binding protein G(I)/G(S)/G(O) subunit gamma-7
map04726	Serotonergic synapse	0.013872	0.200943	3	30	40	2326	Over	A0A024R7J0 Q01970 O60262	Protein kinase, cAMP-dependent, catalytic, alpha, isoform CRA_c,1-phosphatidylinositol 4,5-bisphosphate phosphodiesterase beta-3,Guanine nucleotide-binding protein G(I)/G(S)/G(O) subunit gamma-7
map04725	Cholinergic synapse	0.017991	0.200943	3	33	40	2326	Over	A0A024R7J0 Q01970 O60262	Protein kinase, cAMP-dependent, catalytic, alpha, isoform CRA_c,1-phosphatidylinositol 4,5-bisphosphate phosphodiesterase beta-3,Guanine nucleotide-binding protein G(I)/G(S)/G(O) subunit gamma-7
map05146	Amoebiasis	0.017991	0.200943	3	33	40	2326	Over	P02751 A0A024R7J0 Q01970	Fibronectin,Protein kinase, cAMP-dependent, catalytic, alpha, isoform CRA_c,1-phosphatidylinositol 4,5-bisphosphate phosphodiesterase beta-3
map04750	Inflammatory mediator regulation of TRP channels	0.021078	0.200943	3	35	40	2326	Over	A0A024R7J0 Q01970 A8K1U5	Protein kinase, cAMP-dependent, catalytic, alpha, isoform CRA_c,1-phosphatidylinositol 4,5-bisphosphate phosphodiesterase beta-3,cDNA FLJ75602, highly similar to Homo sapiens amiloride-sensitive cation channel 2, neuronal (ACCN2), transcript variant 2, mRNA
map04915	Estrogen signaling pathway	0.029994	0.252307	3	40	40	2326	Over	A0A024R7J0 Q01970 P08047	Protein kinase, cAMP-dependent, catalytic, alpha, isoform CRA_c,1-phosphatidylinositol 4,5-bisphosphate phosphodiesterase beta-3,Transcription factor Sp1

map04926	Relaxin signaling pathway	0.040617	0.279246	3	45	40	2326	Over	A0A024R7J0 Q01970 O60262	Protein kinase, cAMP-dependent, catalytic, alpha, isoform CRA_c,1-phosphatidylinositol 4,5-bisphosphate phosphodiesterase beta-3,Guanine nucleotide-binding protein G(I)/G(S)/G(O) subunit gamma-7
map04261	Adrenergic signaling in cardiomyocytes	0.052906	0.283732	3	50	40	2326	Over	A0A0S2Z4I4 A0A024R7J0 Q01970	Tropomyosin 3 isoform 3 (Fragment),Protein kinase, cAMP-dependent, catalytic, alpha, isoform CRA_c,1-phosphatidylinositol 4,5-bisphosphate phosphodiesterase beta-3
map04723	Retrograde endocannabinoid signaling	0.052906	0.283732	3	50	40	2326	Over	A0A024R7J0 Q01970 O60262	Protein kinase, cAMP-dependent, catalytic, alpha, isoform CRA_c,1-phosphatidylinositol 4,5-bisphosphate phosphodiesterase beta-3,Guanine nucleotide-binding protein G(I)/G(S)/G(O) subunit gamma-7
map04728	Dopaminergic synapse	0.052906	0.283732	3	50	40	2326	Over	A0A024R7J0 Q01970 O60262	Protein kinase, cAMP-dependent, catalytic, alpha, isoform CRA_c,1-phosphatidylinositol 4,5-bisphosphate phosphodiesterase beta-3,Guanine nucleotide-binding protein G(I)/G(S)/G(O) subunit gamma-7
map04371	Apelin signaling pathway	0.063893	0.283732	3	54	40	2326	Over	A0A024R7J0 Q01970 O60262	Protein kinase, cAMP-dependent, catalytic, alpha, isoform CRA_c,1-phosphatidylinositol 4,5-bisphosphate phosphodiesterase beta-3,Guanine nucleotide-binding protein G(I)/G(S)/G(O) subunit gamma-7
map04062	Chemokine signaling pathway	0.08875	0.333979	3	62	40	2326	Over	A0A024R7J0 Q01970 O60262	Protein kinase, cAMP-dependent, catalytic, alpha, isoform CRA_c,1-phosphatidylinositol 4,5-bisphosphate phosphodiesterase beta-3,Guanine nucleotide-binding protein G(I)/G(S)/G(O) subunit gamma-7
map05225	Hepatocellular carcinoma	0.106081	0.36999	3	67	40	2326	Over	A0A3F2YNW7 E7BJU4 A0A024RAX2	AT-rich interactive domain-containing protein 1B,Heme oxygenase 1 (Fragment),Microsomal glutathione S-transferase 1, isoform CRA_a
map04144	Endocytosis	0.739872	0.846414	3	149	40	2326	Over	Q13596 A0A024R571 A0A024R8B8	Sorting nexin-1,EH domain-containing protein 1,Chromosome 9 open reading frame 28, isoform CRA_a
map04740	Olfactory transduction	0.014381	0.200943	2	11	40	2326	Over	A0A024R7J0 O60262	Protein kinase, cAMP-dependent, catalytic, alpha, isoform CRA_c,Guanine nucleotide-binding protein G(I)/G(S)/G(O) subunit gamma-7
map00670	One carbon pool by folate	0.019958	0.200943	2	13	40	2326	Over	P34897 P13995	Serine hydroxymethyltransferase, mitochondrial,Bifunctional methylenetetrahydrofolate dehydrogenase/cyclohydrolase, mitochondrial
map04924	Renin secretion	0.029724	0.252307	2	16	40	2326	Over	A0A024R7J0 Q01970	Protein kinase, cAMP-dependent, catalytic, alpha, isoform CRA_c,1-phosphatidylinositol 4,5-bisphosphate phosphodiesterase beta-3
map05032	Morphine addiction	0.033325	0.264749	2	17	40	2326	Over	A0A024R7J0 O60262	Protein kinase, cAMP-dependent, catalytic, alpha, isoform CRA_c,Guanine nucleotide-binding protein G(I)/G(S)/G(O) subunit gamma-7

map00250	Alanine, aspartate and glutamate metabolism	0.037089	0.279141	2	18	40	2326	Over	P08243 A0A024R6R2	Asparagine synthetase [glutamine-hydrolyzing],Glutamic pyruvate transaminase (Alanine aminotransferase) 2, isoform CRA_b
map04727	GABAergic synapse	0.041008	0.279246	2	19	40	2326	Over	A0A024R7J0 O60262	Protein kinase, cAMP-dependent, catalytic, alpha, isoform CRA_c,Guanine nucleotide-binding protein G(I)/G(S)/G(O) subunit gamma-7
map00860	Porphyrin and chlorophyll metabolism	0.045077	0.283732	2	20	40	2326	Over	E7BJU4 Q5JPH6	Heme oxygenase 1 (Fragment),Probable glutamate--tRNA ligase, mitochondrial
map04970	Salivary secretion	0.05364	0.283732	2	22	40	2326	Over	A0A024R7J0 Q01970	Protein kinase, cAMP-dependent, catalytic, alpha, isoform CRA_c,1-phosphatidylinositol 4,5-bisphosphate phosphodiesterase beta-3
map00630	Glyoxylate and dicarboxylate metabolism	0.058122	0.283732	2	23	40	2326	Over	P34897 Q8TE01	Serine hydroxymethyltransferase, mitochondrial,DERP12 (Dermal papilla derived protein 12)
map04350	TGF-beta signaling pathway	0.062731	0.283732	2	24	40	2326	Over	Q14766 P08047	Latent-transforming growth factor beta-binding protein 1,Transcription factor Sp1
map04918	Thyroid hormone synthesis	0.067461	0.283732	2	25	40	2326	Over	A0A024R7J0 Q01970	Protein kinase, cAMP-dependent, catalytic, alpha, isoform CRA_c,1-phosphatidylinositol 4,5-bisphosphate phosphodiesterase beta-3
map04961	Endocrine and other factor-regulated calcium reabsorption	0.067461	0.283732	2	25	40	2326	Over	A0A024R7J0 Q01970	Protein kinase, cAMP-dependent, catalytic, alpha, isoform CRA_c,1-phosphatidylinositol 4,5-bisphosphate phosphodiesterase beta-3
map05414	Dilated cardiomyopathy (DCM)	0.067461	0.283732	2	25	40	2326	Over	A0A0S2Z4I4 A0A024R7J0	Tropomyosin 3 isoform 3 (Fragment),Protein kinase, cAMP-dependent, catalytic, alpha, isoform CRA_c
map04971	Gastric acid secretion	0.072306	0.295422	2	26	40	2326	Over	A0A024R7J0 Q01970	Protein kinase, cAMP-dependent, catalytic, alpha, isoform CRA_c,1-phosphatidylinositol 4,5-bisphosphate phosphodiesterase beta-3
map04916	Melanogenesis	0.082324	0.31817	2	28	40	2326	Over	A0A024R7J0 Q01970	Protein kinase, cAMP-dependent, catalytic, alpha, isoform CRA_c,1-phosphatidylinositol 4,5-bisphosphate phosphodiesterase beta-3
map04925	Aldosterone synthesis and secretion	0.082324	0.31817	2	28	40	2326	Over	A0A024R7J0 Q01970	Protein kinase, cAMP-dependent, catalytic, alpha, isoform CRA_c,1-phosphatidylinositol 4,5-bisphosphate phosphodiesterase beta-3
map00970	Aminoacyl-tRNA biosynthesis	0.10353	0.36999	2	32	40	2326	Over	A0A0S2Z4R1 Q5JPH6	Tyrosine--tRNA ligase (Fragment),Probable glutamate--tRNA ligase, mitochondrial
map04720	Long-term potentiation	0.10353	0.36999	2	32	40	2326	Over	A0A024R7J0 Q01970	Protein kinase, cAMP-dependent, catalytic, alpha, isoform CRA_c,1-phosphatidylinositol 4,5-bisphosphate phosphodiesterase beta-3
map00562	Inositol phosphate metabolism	0.109048	0.371283	2	33	40	2326	Over	Q01970 A0A024RC06	1-phosphatidylinositol 4,5-bisphosphate phosphodiesterase beta-3,Myotubularin 1, isoform CRA_a

map04540	Gap junction	0.131863	0.4012	2	37	40	2326	Over	A0A024R7J0 Q01970	Protein kinase, cAMP-dependent, catalytic, alpha, isoform CRA_c,1-phosphatidylinositol 4,5-bisphosphate phosphodiester-ase beta-3
map04912	GnRH signaling pathway	0.131863	0.4012	2	37	40	2326	Over	A0A024R7J0 Q01970	Protein kinase, cAMP-dependent, catalytic, alpha, isoform CRA_c,1-phosphatidylinositol 4,5-bisphosphate phosphodiester-ase beta-3
map04020	Calcium signaling pathway	0.137732	0.410326	2	38	40	2326	Over	A0A024R7J0 Q01970	Protein kinase, cAMP-dependent, catalytic, alpha, isoform CRA_c,1-phosphatidylinositol 4,5-bisphosphate phosphodiester-ase beta-3
map01522	Endocrine resistance	0.143659	0.414055	2	39	40	2326	Over	A0A024R7J0 P08047	Protein kinase, cAMP-dependent, catalytic, alpha, isoform CRA_c,Transcription factor Sp1
map04270	Vascular smooth muscle contraction	0.149642	0.419584	2	40	40	2326	Over	A0A024R7J0 Q01970	Protein kinase, cAMP-dependent, catalytic, alpha, isoform CRA_c,1-phosphatidylinositol 4,5-bisphosphate phosphodiester-ase beta-3
map04070	Phosphatidylinositol signaling system	0.161757	0.420569	2	42	40	2326	Over	Q01970 A0A024RC06	1-phosphatidylinositol 4,5-bisphosphate phosphodiesterase beta-3,Myotubularin 1, isoform CRA_a
map00010	Glycolysis / Gluco-neogenesis	0.167884	0.428703	2	43	40	2326	Over	Q2TSD0 Q8TE01	Glyceraldehyde-3-phosphate dehydrogenase,DERP12 (Dermal papilla derived protein 12)
map04933	AGE-RAGE signal-ing pathway in dia-betic complications	0.180258	0.448843	2	45	40	2326	Over	P02751 Q01970	Fibronectin,1-phosphatidylinositol 4,5-bisphosphate phos-phodiesterase beta-3
map04310	Wnt signaling path-way	0.186499	0.448843	2	46	40	2326	Over	A0A024R7J0 Q01970	Protein kinase, cAMP-dependent, catalytic, alpha, isoform CRA_c,1-phosphatidylinositol 4,5-bisphosphate phosphodiester-ase beta-3
map04922	Glucagon signaling pathway	0.192773	0.451911	2	47	40	2326	Over	A0A024R7J0 Q01970	Protein kinase, cAMP-dependent, catalytic, alpha, isoform CRA_c,1-phosphatidylinositol 4,5-bisphosphate phosphodiester-ase beta-3
map04066	HIF-1 signaling pathway	0.205407	0.458956	2	49	40	2326	Over	Q2TSD0 E7BJU4	Glyceraldehyde-3-phosphate dehydrogenase,Heme oxygenase 1 (Fragment)
map05418	Fluid shear stress and atherosclerosis	0.218137	0.465575	2	51	40	2326	Over	E7BJU4 A0A024RAX2	Heme oxygenase 1 (Fragment),Microsomal glutathione S-trans-ferase 1, isoform CRA_a
map04921	Oxytocin signaling pathway	0.25025	0.48197	2	56	40	2326	Over	A0A024R7J0 Q01970	Protein kinase, cAMP-dependent, catalytic, alpha, isoform CRA_c,1-phosphatidylinositol 4,5-bisphosphate phosphodiester-ase beta-3
map04611	Platelet activation	0.263162	0.48873	2	58	40	2326	Over	A0A024R7J0 Q01970	Protein kinase, cAMP-dependent, catalytic, alpha, isoform CRA_c,1-phosphatidylinositol 4,5-bisphosphate phosphodiester-ase beta-3
map04360	Axon guidance	0.28255	0.505058	2	61	40	2326	Over	A8K139 B4E2I9	cDNA FLJ76744, highly similar to Homo sapiens L1 cell adhe-sion molecule (L1CAM), transcript variant 1, mRNA,cDNA FLJ55486, highly similar to Semaphorin-3C

map04919	Thyroid hormone signaling pathway	0.28901	0.510228	2	62	40	2326	Over	A0A024R7J0 Q01970	Protein kinase, cAMP-dependent, catalytic, alpha, isoform CRA_c,1-phosphatidylinositol 4,5-bisphosphate phosphodiesterase beta-3
map04014	Ras signaling pathway	0.372026	0.596838	2	75	40	2326	Over	A0A024R7J0 O60262	Protein kinase, cAMP-dependent, catalytic, alpha, isoform CRA_c,Guanine nucleotide-binding protein G(I)/G(S)/G(O) subunit gamma-7
map05010	Alzheimer's disease	0.39693	0.616214	2	79	40	2326	Over	Q2TSD0 Q01970	Glyceraldehyde-3-phosphate dehydrogenase,1-phosphatidylinositol 4,5-bisphosphate phosphodiesterase beta-3
map04530	Tight junction	0.646734	0.770692	2	80	40	2326	Over	P35580 A0A024R7J0	Myosin-10,Protein kinase, cAMP-dependent, catalytic, alpha, isoform CRA_c
map05205	Proteoglycans in cancer	0.656158	0.775459	2	85	40	2326	Over	P02751 A0A024R7J0	Fibronectin,Protein kinase, cAMP-dependent, catalytic, alpha, isoform CRA_c
map05016	Huntington's disease	0.675419	0.79168	2	94	40	2326	Over	Q01970 P08047	1-phosphatidylinositol 4,5-bisphosphate phosphodiesterase beta-3,Transcription factor Sp1
map04151	PI3K-Akt signaling pathway	0.691784	0.804269	2	101	40	2326	Over	P02751 O60262	Fibronectin,Guanine nucleotide-binding protein G(I)/G(S)/G(O) subunit gamma-7
map05165	Human papillomavirus infection	0.718757	0.82889	2	112	40	2326	Over	P02751 A0A024R7J0	Fibronectin,Protein kinase, cAMP-dependent, catalytic, alpha, isoform CRA_c
map00750	Vitamin B6 metabolism	0.067076	0.283732	1	4	40	2326	Over	B4DHQ3	Phosphoserine aminotransferase
map04742	Taste transduction	0.067076	0.283732	1	4	40	2326	Over	A0A024R7J0	Protein kinase, cAMP-dependent, catalytic, alpha, isoform CRA_c
map00061	Fatty acid biosynthesis	0.114483	0.37207	1	7	40	2326	Over	B2RBE0	cDNA, FLJ95462, highly similar to Homo sapiens fatty-acid-Coenzyme A ligase, long-chain 3 (FACL3),mRNA
map04913	Ovarian steroidogenesis	0.114483	0.37207	1	7	40	2326	Over	A0A024R7J0	Protein kinase, cAMP-dependent, catalytic, alpha, isoform CRA_c
map05143	African trypanosomiasis	0.129757	0.4012	1	8	40	2326	Over	Q01970	1-phosphatidylinositol 4,5-bisphosphate phosphodiesterase beta-3
map00511	Other glycan degradation	0.144774	0.414055	1	9	40	2326	Over	P20933	N(4)-(beta-N-acetylglucosaminyl)-L-asparaginase
map00220	Arginine biosynthesis	0.159539	0.420569	1	10	40	2326	Over	A0A024R6R2	Glutamic pyruvate transaminase (Alanine aminotransferase) 2, isoform CRA_b
map04514	Cell adhesion molecules (CAMs)	0.159539	0.420569	1	10	40	2326	Over	A8K139	cDNA FLJ76744, highly similar to Homo sapiens L1 cell adhesion molecule (L1CAM), transcript variant 1, mRNA
map05030	Cocaine addiction	0.159539	0.420569	1	10	40	2326	Over	A0A024R7J0	Protein kinase, cAMP-dependent, catalytic, alpha, isoform CRA_c
map04340	Hedgehog signaling pathway	0.188326	0.448843	1	12	40	2326	Over	A0A024R7J0	Protein kinase, cAMP-dependent, catalytic, alpha, isoform CRA_c
map01523	Antifolate resistance	0.202356	0.458956	1	13	40	2326	Over	P34897	Serine hydroxymethyltransferase, mitochondrial

map05020	Prion diseases	0.202356	0.458956	1	13	40	2326	Over	A0A024R7J0	Protein kinase, cAMP-dependent, catalytic, alpha, isoform CRA_c
map00982	Drug metabolism - cytochrome P450	0.21615	0.465575	1	14	40	2326	Over	A0A024RAX2	Microsomal glutathione S-transferase 1, isoform CRA_a
map04976	Bile secretion	0.21615	0.465575	1	14	40	2326	Over	A0A024R7J0	Protein kinase, cAMP-dependent, catalytic, alpha, isoform CRA_c
map00983	Drug metabolism - other enzymes	0.243044	0.476101	1	16	40	2326	Over	Q9HA47	Uridine-cytidine kinase 1
map01210	2-Oxocarboxylic acid metabolism	0.243044	0.476101	1	16	40	2326	Over	A0A024R6R2	Glutamic pyruvate transaminase (Alanine aminotransferase) 2, isoform CRA_b
map04923	Regulation of lipoly-sis in adipocytes	0.243044	0.476101	1	16	40	2326	Over	A0A024R7J0	Protein kinase, cAMP-dependent, catalytic, alpha, isoform CRA_c
map04974	Protein digestion and absorption	0.243044	0.476101	1	16	40	2326	Over	P25940	Collagen alpha-3(V) chain
map05031	Amphetamine addic-tion	0.243044	0.476101	1	16	40	2326	Over	A0A024R7J0	Protein kinase, cAMP-dependent, catalytic, alpha, isoform CRA_c
map05204	Chemical carcino-genesis	0.243044	0.476101	1	16	40	2326	Over	A0A024RAX2	Microsomal glutathione S-transferase 1, isoform CRA_a
map00980	Metabolism of xeno-biotics by cyto-chrome P450	0.256152	0.48197	1	17	40	2326	Over	A0A024RAX2	Microsomal glutathione S-transferase 1, isoform CRA_a
map04115	p53 signaling path-way	0.256152	0.48197	1	17	40	2326	Over	Q658P3	Metalloreductase STEAP3
map03410	Base excision repair	0.269038	0.493236	1	18	40	2326	Over	P78549	Endonuclease III-like protein 1
map04512	ECM-receptor inter-action	0.281706	0.505058	1	19	40	2326	Over	P02751	Fibronectin
map00480	Glutathione metabo-lism	0.330273	0.575964	1	23	40	2326	Over	A0A024RAX2	Microsomal glutathione S-transferase 1, isoform CRA_a
map04962	Vasopressin-regu-lated water reabsorp-tion	0.341905	0.589066	1	24	40	2326	Over	A0A024R7J0	Protein kinase, cAMP-dependent, catalytic, alpha, isoform CRA_c
map04260	Cardiac muscle con-traction	0.35334	0.594443	1	25	40	2326	Over	A0A0S2Z4I4	Tropomyosin 3 isoform 3 (Fragment)
map04972	Pancreatic secretion	0.35334	0.594443	1	25	40	2326	Over	Q01970	1-phosphatidylinositol 4,5-bisphosphate phosphodiesterase beta-3
map00640	Propanoate metabo-lism	0.364582	0.596838	1	26	40	2326	Over	Q8TE01	DERP12 (Dermal papilla derived protein 12)
map05410	Hypertrophic cardio-myopathy (HCM)	0.364582	0.596838	1	26	40	2326	Over	A0A0S2Z4I4	Tropomyosin 3 isoform 3 (Fragment)

map03320	PPAR signaling pathway	0.375632	0.596838	1	27	40	2326	Over	B2RBE0	cDNA, FLJ95462, highly similar to Homo sapiens fatty-acid-Coenzyme A ligase, long-chain 3 (FACL3),mRNA
map04730	Long-term depression	0.375632	0.596838	1	27	40	2326	Over	Q01970	1-phosphatidylinositol 4,5-bisphosphate phosphodiesterase beta-3
map00620	Pyruvate metabolism	0.407672	0.616214	1	30	40	2326	Over	Q8TE01	DERP12 (Dermal papilla derived protein 12)
map05110	Vibrio cholerae infection	0.407672	0.616214	1	30	40	2326	Over	A0A024R7J0	Protein kinase, cAMP-dependent, catalytic, alpha, isoform CRA_c
map05230	Central carbon metabolism in cancer	0.407672	0.616214	1	30	40	2326	Over	Q01650	Large neutral amino acids transporter small subunit 1
map00071	Fatty acid degradation	0.417992	0.616214	1	31	40	2326	Over	B2RBE0	cDNA, FLJ95462, highly similar to Homo sapiens fatty-acid-Coenzyme A ligase, long-chain 3 (FACL3),mRNA
map01212	Fatty acid metabolism	0.417992	0.616214	1	31	40	2326	Over	B2RBE0	cDNA, FLJ95462, highly similar to Homo sapiens fatty-acid-Coenzyme A ligase, long-chain 3 (FACL3),mRNA
map04721	Synaptic vesicle cycle	0.417992	0.616214	1	31	40	2326	Over	P20336	Ras-related protein Rab-3A
map01524	Platinum drug resistance	0.428136	0.618418	1	32	40	2326	Over	A0A024RAX2	Microsomal glutathione S-transferase 1, isoform CRA_a
map04920	Adipocytokine signaling pathway	0.428136	0.618418	1	32	40	2326	Over	B2RBE0	cDNA, FLJ95462, highly similar to Homo sapiens fatty-acid-Coenzyme A ligase, long-chain 3 (FACL3),mRNA
map00020	Citrate cycle (TCA cycle)	0.438107	0.626493	1	33	40	2326	Over	Q8TE01	DERP12 (Dermal papilla derived protein 12)
map04137	Mitophagy - animal	0.457544	0.635231	1	35	40	2326	Over	P08047	Transcription factor Sp1
map04213	Longevity regulating pathway - multiple species	0.457544	0.635231	1	35	40	2326	Over	A0A024R7J0	Protein kinase, cAMP-dependent, catalytic, alpha, isoform CRA_c
map04914	Progesterone-mediated oocyte maturation	0.457544	0.635231	1	35	40	2326	Over	A0A024R7J0	Protein kinase, cAMP-dependent, catalytic, alpha, isoform CRA_c
map05142	Chagas disease (American trypanosomiasis)	0.467015	0.642146	1	36	40	2326	Over	Q01970	1-phosphatidylinositol 4,5-bisphosphate phosphodiesterase beta-3
map05224	Breast cancer	0.476325	0.648709	1	37	40	2326	Over	P08047	Transcription factor Sp1
map05222	Small cell lung cancer	0.485476	0.654935	1	38	40	2326	Over	P02751	Fibronectin
map04146	Peroxisome	0.503313	0.66031	1	40	40	2326	Over	B2RBE0	cDNA, FLJ95462, highly similar to Homo sapiens fatty-acid-Coenzyme A ligase, long-chain 3 (FACL3),mRNA
map05034	Alcoholism	0.503313	0.66031	1	40	40	2326	Over	O60262	Guanine nucleotide-binding protein G(I)/G(S)/G(O) subunit gamma-7
map05231	Choline metabolism in cancer	0.503313	0.66031	1	40	40	2326	Over	P08047	Transcription factor Sp1

map00280	Valine, leucine and isoleucine degradation	0.512004	0.665605	1	41	40	2326	Over	Q8TE01	DERP12 (Dermal papilla derived protein 12)
map04211	Longevity regulating pathway	0.520546	0.670614	1	42	40	2326	Over	A0A024R7J0	Protein kinase, cAMP-dependent, catalytic, alpha, isoform CRA_c
map05202	Transcriptional misregulation in cancer	0.553282	0.706423	1	46	40	2326	Over	P08047	Transcription factor Sp1
map04072	Phospholipase D signaling pathway	0.561119	0.707318	1	47	40	2326	Over	Q01970	1-phosphatidylinositol 4,5-bisphosphate phosphodiesterase beta-3
map00240	Pyrimidine metabolism	0.568822	0.707318	1	48	40	2326	Over	Q9HA47	Uridine-cytidine kinase 1
map04931	Insulin resistance	0.568822	0.707318	1	48	40	2326	Over	X6R3N0	Solute carrier family 27 (Fatty acid transporter), member 3, isoform CRA_d
map04022	cGMP-PKG signaling pathway	0.591149	0.722516	1	51	40	2326	Over	Q01970	1-phosphatidylinositol 4,5-bisphosphate phosphodiesterase beta-3
map05100	Bacterial invasion of epithelial cells	0.591149	0.722516	1	51	40	2326	Over	P02751	Fibronectin
map04024	cAMP signaling pathway	0.605403	0.733666	1	53	40	2326	Over	A0A024R7J0	Protein kinase, cAMP-dependent, catalytic, alpha, isoform CRA_c
map04114	Oocyte meiosis	0.619172	0.744047	1	55	40	2326	Over	A0A024R7J0	Protein kinase, cAMP-dependent, catalytic, alpha, isoform CRA_c
map04071	Sphingolipid signaling pathway	1	1	1	57	40	2326	Over	Q01970	1-phosphatidylinositol 4,5-bisphosphate phosphodiesterase beta-3
map04142	Lysosome	1	1	1	56	40	2326	Over	P20933	N(4)-(beta-N-acetylglucosaminyl)-L-asparaginase

Table S3. Targets associated with Alzheimer's disease from different databases.

Gene Symbol
ACE
APP
ADAM10
GSK3B
HFE
APOE
MAPT
TREM2
PSEN1
PLAU
BACE1
IDE
IL1B
INSR
LEP
NPY
BCL2
BDNF
CASP3
IGF2
IGF1R
ATP5F1A
INS
BAX
ABCA7
TOMM40
CLU
CR1
A2M
EPHA1
CD2AP
BIN1
APOC1
MPO
NOS3
PSEN2
SORL1
PICALM
VSNL1
INPP5D
NECTIN2
MS4A4A
PCDH11X
CASS4
CYP46A1
CHRNA7
CST3
CYP2D6
DHCR24
DPYSL2
ESR1
NCSTN
HMOX1
IGF1
IL6
MIR146A
MAOB
ACHE
MTHFR

PPARG
PRNP
RELN
BCHE
TFAM
TNF
VEGFA
CD33
CRH
SOD2
PLCG2
UNC5C
ABI3
WWOX
TF
CHRNB2
SLC30A6
PGRMC1
EIF2S1
F2
ARC
CALM1
ENO1
HLA-DRB5
IGF2R
TPI1
AMFR
MIR100
MIR375
SLC2A4
SLC30A4
MIR708
CDK5
PPARGC1A
CHAT
TPP1
MAPK14
CTNNB1
AGER
GAPDH
GAPDHS
IRS1
LRP1
MAP2
MME
NFE2L2
NGF
NGFR
NOS2
PIN1
PYY
PTGS2
S100B
SOD1
SYP
ADAMTS1
NTRK2
CAV1
PTGS1
DNM1
APLP2
ADAM17

MFN2
HSF1
CCR5
GSR
HSPD1
IL33
CIB1
HSPB1
CASP8
IKBKB
SERPINF1
MT2A
ATP7A
BCL2L2
CASP9
ADAM9
CASP12
FERMT2
ABCA1
PTK2B
PLCB1
GRN
SQSTM1
GAB2
HLA-DRB1
NFIC
CSF1R
HSPG2
APOB
MARK4
MS4A6A
CELF1
VCP
SYNJ1
ZCWPW1
F13A1
APOC2
MS4A4E
APH1B
EXOC3L2
AKAP9
PILRA
LAMP1
FRMD4A
DST
MADD
PLXNA4
ADAMTS4
SPON1
SLC24A4
GLIS3
MTHFD1L
IL6R
DCHS2
AICDA
BCL3
SPI1
TGFB2
COL18A1
MEGF10
TRIP4
CDH13

CELF2
NDUFAF6
AP2A2
EPHX2
SORCS3
LAMA1
RBFOX1
FMN2
PTPRG
EXOC4
SGK1
ST6GAL1
TCF7L2
UBE2L3
ZAP70
SPPL2A
SUCLG2
ALDH1A2
HDAC6
BACE1-AS
DNM1L
GPC6
G3BP1
FARP1
TSHZ1
DCAF7
SIGMAR1
BCKDK
RTN3
CDR1
AHSA1
KHDRBS1
CETP
STAG3
TPPP
AKR1C4
CLASRP
CHI3L1
PSIP1
CHRNA2
CHRNA4
NLRP3
SERPINA3
LRRK2
AGBL1
ACKR2
CNR2
COL4A4
COMT
CP
CREB1
CRK
CRP
PARP1
CSF2
CTNNA2
CTSB
CTSD
CYP8B1
CYP19A1
DAPK1
DBN1

DLG4
DPP4
RCAN1
AGT
DYRK1A
AIF1
ABCA2
SPRED2
ENO2
C9orf72
EPHB2
EPO
AKT1
ESR2
ALB
ALDH2
FANCD2
SIRT2
DKK1
ZNF292
WWC1
SIRT3
SIRT1
NCS1
TARDBP
NUP62
PLD3
ALOX5
STH
MTOR
FYN
CALHM1
GABRA2
PCSK9
GABRG3
QPCT
BACE2
RNF19A
GAP43
CNTNAP2
TRPC4AP
GATA1
GCG
GDNF
GFAP
GLP1R
GRIN2B
NR3C1
GRM5
GULOP
UBQLN1
ANXA1
HSD17B10
HCRT
CFH
APBB1
HSPA4
HSP90AA1
APOA1
HTR2A
HTR6
IAPP

TGM6
IFNG
APOC4
APOD
FAS
IL1A
IL4
CXCL8
IL10
IL12A
IL17A
IL18
AQP4
KCNN2
KNG1
BLOC1S3
RPSA
LCN2
LDLR
LPA
LPL
LTBP2
BCAM
MIR107
MIR132
MIR29A
MIR34A
MAOA
ARVCF
TRPM1
MMP9
MOBP
MT3
NEFL
NOS1
NPC1
NRGN
OGG1
SERPINE1
APH1A
DCTN4
WAC
ABCB1
PIK3CA
PIK3CB
PIK3CG
PLA2G1B
PLG
IL6-AS1
CYCS
POLD1
PON1
PPARA
BCAS3
CDKAL1
RMDN3
PTPA
STK32B
PRKAA1
PRKAA2
PRKAB1
PRKCA

PRKCB
MAPK1
MAPK8
EIF2AK2
PTPRA
PVALB
PVR
RAC1
RELB
REN
REST
ACTB
SORT1
CCL2
CX3CL1
CSMD1
PINK1
SLC1A2
SLC6A3
SLC6A4
SNAP25
SNCB
SNCA
SNCG
SOAT1
SST
TCF3
TGFB1
TH
TSPO
THY1
TLR2
TLR4
ACTG1
TNFRSF1B
TP53
C3
ACTG2
TTR
TYROBP
UBB
UCHL1
VDR
VLDLR
YWHAZ
AHNAK
CALB1
TFEB
AIMP2
PPP1R3B
ANKRD55
GEMIN7
CLMN
EHMT1
THSD4
SP6
CAPN1
CASP1
CASP6
KAT8
CAT
AGPS

KHSRP
RUNX1T1
PDE5A
BECN1
CRADD
SUCLA2
CDK5R1
CACNA1G
MPZL1
CCRL2
AP4M1
LRAT
CD14
KL
ADIPOQ
LIPG
ITM2B
GSTO1
CD36
SCARB1
CLOCK
CD40
CD68
HDAC9
CDK1
ELMO1
MVP
PRRT2
SORCS1
EGR1
GSN
HDAC2
IL2
ITGAM
OLR1
P2RX7
PDE4A
ATP7B
MAPK3
KLK6
RAB5A
S100A9
CCL11
BLMH
POTEF
SUMO1
VDAC1
LRP8
COL25A1
CASR
NR1I2
CSNK1D
DBH
DLST
AGTR1
NLRP1
ANK1
HTT
NRG1
HMGCR
HSD11B1
APLP1

IL1RN
IL13
AR
ACAT1
LRP2
MIR155
NFIA
NFIB
NTRK1
OGDH
PAEP
GAL
PLA2G4A
VPS35
PSENEN
REG1A
SET
BLVRA
SOX2
STAT3
TFCP2
XBP1
FZD4
KAT5
OGA
CNTF
RBM45
CREBBP
ECE1
FGF2
ADNP
FPR2
CHMP2B
GRIA1
GRIA2
CTNNA3
GSTM1
KCNIP3
HIF1A
APBA1
HP
APCS
ICAM1
HSD17B13
APRT
KLC1
LBP
MIR206
MMP3
NFKB1
NOTCH1
NTF3
PAWR
SERPINA1
GSAP
TMEM106B
ST3GAL4
BRCA1
TFRC
TNFRSF1A
C4A
C4B

DHRS11
PLA2G6
TTBK1
RIPK1
ABCG2
EIF2AK3
CD44
ABCG1
PPIF
TMED10
KLK8
CPOX
CRHR1
CSF3
CX3CR1
CYP3A4
FGF1
FKBP4
FKBP5
FOXO3
FLT1
FUS
GJA1
ANPEP
GRIN2A
GSTP1
HMGA1
HSPA1A
IGFBP3
CXCL10
MIR29C
MARK1
MEFV
MIF
MYC
SLC11A2
PHF1
PLA2G2A
PON2
PREP
SLC17A7
S100A1
SP1
SREBF2
SYK
C5AR1
VCAM1
CALB2
CAST
SHANK3
CH25H
XPR1
COX5A
CD40LG
CDC42
NAT2
RANBP9
ABCC9
TUBA1B
CEBPB
CEBPD
TXNIP

NES
CPLX1
GSTO2
CNR1
CRYAB
CCN2
ADRB2
DAB1
DECR1
NQO1
DLD
DNAH8
EDN1
EEF2
EGFR
ELANE
ELAVL2
EPHA4
ETS2
F2R
FN1
FOS
ABCA4
GDF2
GH1
HTRA2
GLUL
GPR3
GRM2
GSTT1
HLA-A
HMGB1
HSPA1B
HSPA5
HSPA8
IGFBP2
MIR137
MIR21
MIR212
MIR29B1
MEF2C
MMP2
MMP14
MSMB
ATM
NEDD9
P4HB
PRKN
NME8
IL23A
PECAM1
CALML5
PLTP
TREM1
MAPK10
PSPN
LGMN
HTRA1
PTBP1
PTEN
NDRG2
PTPN1

HAMP
PTPRC
BCYRN1
MIR455
RTN4R
SLC2A1
SLC2A3
SPTBN1
SYN1
SYT1
PRDX2
TGM2
TIMP1
SERPING1
VIM
XRCC1
DDR1
RAB7A
PLA2G7
LIN28A
TP63
CBS
APLN
KALRN
NTN1
ROCK2
CD59
OPTN
CDKN2A
HPSE
RAB10
ADCYAP1
COL11A2
ADM
ADORA2A
ADRA2B
CYP2B6
CYP2C9
GRK2
CD55
DAO
DCX
DDIT3
DIO2
DRD1
DRD4
AGTR2
EIF4E
CRTC1
FOLH1
SRRM2
FXN
ALOX15
FNDC5
GAD1
GBA
DISC1
NEAT1
GPT
GZMB
UBE2K
HNRNPA1

APBB2
HSPA9
HSPB2
HTR4
APOA4
IFNA1
APOC3
LRRTM3
KLK3
IL9
AQP1
IDO1
ITGAX
ITPR3
LGALS3
LIPA
LRP6
ARNTL
MIR106B
MIR142
MIR222
MFGE8
MELTF
MTR
ATF4
NOTCH3
NPTX2
NR4A2
MIR424
P2RY2
PRDX1
PDE4D
PDE9A
PDGFRB
PLD1
TLR9
POLB
POMC
MCOLN1
STIM2
BCL2A1
ATXN1
CCL5
SRR
CXCL12
ABCG4
SELENOP
SH3GL2
WNK1
BMP4
SLC18A2
SLC18A3
SRPK2
BSG
TBP
TRPC6
UGCG
NR1H2
VGF
XPNPEP1
SLC30A3
CXCR4

FTO
MMEL1
TREML2
COASY
CALM3
CAMK2A
MAP1LC3B
OGT
ATCAY
IRS2
MBTPS1
HDAC3
WNT3A
HDAC4
KEAP1
AD11
CDK4
GPHN
STUB1
AKR1A1
SLC9A6
PITRM1
NCKAP1
CPLX2
EDAR
IMMT
CIT
CHGA
CHRM1
CHRM2
CKB
ATF2
CS
CSF1
ADRA1A
CTRL
CTSS
ADRB1
CYBB
CYP17A1
DNM2
DNMT3B
DRD3
EDNRA
EGF
ERBB4
AKT2
F2RL1
F12
FAAH
FABP3
FCGR3A
FDPS
FGFR3
NLGN1
SHANK2
FLNA
DNMBP
PRND
PPP1R15A
CIZ1
GAS6

GC
AATF
GLB1
GLO1
ANGPT1
GPX1
GRB2
GRIN1
PYCARD
CD274
CXCL1
GSTM3
SLC40A1
ANXA5
HK1
HMGCS2
APBA2
HPS1
APEX1
HTR2C
IGFBP7
INPPL1
ITPR1
JUN
MALAT1
L1CAM
LAMP2
LHCGR
LIF
LMNA
LMNB1
CHCHD10
LOX
LYZ
MIR181C
MIR188
MIR200A
MIR200B
MIR26B
ARRB2
MBP
MECP2
MEOX2
MYD88
NEFM
NEUROD1
ACO1
NPPA
NTS
OPRD1
OPRK1
PCK1
PCNA
CHCHD2
GOLM1
PER1
DDIT4
PPARD
AVP
RCBTB1
PPP3R1
MAP2K1

MAP2K2
PRL
CTNNBL1
PROS1
RETN
BAG1
PTGDS
ACE2
RBP4
RELA
RGS2
RGS4
ROCK1
RPS6
RPS6KB1
RXRA
RYR2
RYR3
SAA1
ATXN2
SCD
CCL3
SFPQ
SGCA
SHBG
SLC6A2
SPP1
TAC1
TAP2
TAT
CNTN2
TERT
TGFBR2
TIMP2
TLE1
TP73
TPH1
TPT1
TSC2
CCR2
TXN
BEST1
TRPV1
XK
YY1
CAD
CALCA
ECHDC3
RIN3
CALM2
CAPN2
CASP2
CASP4
PPP1R1B
SYVN1
TNFSF10
SPHK1
PPIG
NRXN3
MAPK8IP1
BAG3
PTGES

APBA3
PRDX6
CARTPT
CD69
SNAP91
SV2A
CDH1
BCL2L11
NR1H3
LRPPRC
FLOT1
CDKN1A
CDKN1B
CDR1-AS
KLF2
CPQ
RAPGEF3
LRP1-AS
CES1
CTCF
CFL2
CGA
CGB3
PDE10A
LILRB1
ADAMTS13
CHEK1
CHIT1
CHEK2
CHM
PADI2
HRH3
PHB2
MGLL
AZIN2
CHRNA3
GPRASP2
APOA5
ADD3
TPH2
CCR3
ADH1B
HSPB6
COL17A1
COX10
CPE
CSNK1E
TTBK2
CTF1
CTNND2
CTSG
CTSZ
CXADR
CYP1A2
GADD45A
AEBP1
DLG2
DLG3
DMD
DNMT1
DOCK2
ATN1

DUSP1
DUSP6
E2F1
AHSG
MLKL
ELK1
ELN
EP300
ERN1
ALAS1
F11
FABP5
BPTF
FGF14
FKBP1A
NMNAT2
FOXO1
FLG
ASTN2
SYNE1
ALPP
MSRB3
SH2B1
PTCD1
APPL1
AMD1
HSPB8
GGT1
PDCD4
AMPH
PCSK1N
GLS
ABO
GPC1
GPI
IFNL3
CXCR3
DLL1
GRIA3
SETD2
TBK1
NCAPH2
HCRTR2
HDAC1
HGF
HHEX
ANXA6
HLA-B
HMOX2
HNMT
APC
APEH
HTR1A
HTR1B
APOA2
HTR7
RAB7B
TMEM119
IL1R1
IL1RAP
IL6ST
CXCR2

IL15
IL16
ILK
ITGAL
ITGB1
ITGB2
ITIH4
KCNB1
AGRN
KDR
ARG1
RHOA
ARMS2
STMN1
LIPC
LNPEP
MIRLET7B
MIR125A
MIR144
MIR15B
MIR195
MIR214
ARR3
MIR219A1
MIR22
MIR98
SMAD2
ARSA
MARK3
MBL2
MCL1
CD46
MEF2A
MAP3K5
MET
MICB
MMP1
CD200
ASL
MPZ
ABCC1
EIF2AK4
MIR326
MIR339
MSI1
MTHFD1
MTNR1A
ZFHX3
NCAM1
NCL
NEFH
ATP12A
NOTCH4
PNP
NPM1
NTF4
CISD2
ODC1
OPRL1
OPRM1
OTC
P2RX4

PEBP1
PLA2G3
PAK1
GEMIN4
REG3A
ASCC1
FIS1
ADIPOR1
PCSK1
PDCD1
SIRT6
PGF
SERPINB6
PI4KA
PKM
SERPINF2
PLK1
PNMT
PON3
POU2F1
PPIA
AHI1
TRPM7
PPP1CA
ACP3
PPP1R10
PPP2CA
PPP2R2B
PPP3CA
MAPK9
PRSS3
MASP1
AZU1
SPHK2
PSMB9
PDSS2
PTGER3
MIR193B
KIDINS220
ALS2
TRIB3
NLRC4
PZP
RAB3A
RAB4A
RAB6A
RAP1A
IL21
CCND1
RFC1
S100A6
S100A12
CFB
CCL4
SDC2
SEL1L
SRSF2
SGCG
ITSN1
BMI1
ST8SIA1
SIM2

SLC1A3
BMP6
SLC19A1
SLPI
SMPD1
SPARC
SPAST
SPG7
ST13
STAT1
SULT2A1
BST1
SYN2
TGFBR1
TGM1
TIA1
TLR5
C1QA
C1R
C3AR1
TTPA
TXNRD1
TYK2
TYR
TYRO3
TYRP1
UNG
USF1
USF2
VIP
VWF
WNT1
CA2
SLC30A1
AD10
CALCR
NR4A3
TET1
NPL
CALR
CAMK4
SNX27
CAMP
USP9X
ARHGAP24
CASP7
PPP1R9B
PTPN5
TNK1
CFLAR
CCK
PER3
PER2
WASF1
CHRFAM7A
HAP1
SOCS3
MCU
IL1RL1
ARHGEF2
KLF4
CGB5

FTMT
CD80
OPN4
HOMER1
ATG5
GDF15
NPEPPS
VPS26A
CD47
CLSTN3
NCAPD2
NR1I3
ADA
CDH2
CDKN2B-AS1
KCNE3
MIR873
AD14
GNE
PARK16
MTRNR2L12
OCLN
MICA
PQBP1
CERT1
NOTCH2NLC
BCAP31
LINC01080
OLIG2
CALCOCO2
CDK9
ABCC4
APBB3
TCIRG1
LAMC3
TLR6
CCL26
WARS2
SEMA3A
TUBB4B
RACK1
ADARB1
PEMT
IFITM3
YAP1
GPNMB
ATP5PD
SEMA4D
HYOU1
NPC2
TXNRD2
CAMKK2
NRG3
CFL1
CHL1
CFTR
GJB6
HSPH1
PRDX3
COPS5
RAPGEF4
STMN2

RER1
FAF1
CHGB
CORO1A
WDR45
STX1B
TREX1
CHRM3
PARK7
ECD
SYNPO
OMA1
CISH
GRIN3A
GRIN3B
CLN3
CLN5
LRIG3
H4-16
FRMD6
PLIN2
CCR6
ABCC2
CNP
CNTFR
CNTN1
ACMSD
SLC31A1
ADORA1
COX6B1
COX15
CPN1
CPS1
CPT1A
CSF3R
CSNK2A1
VCAN
CSPG4
PM20D1
GLIS1
IL23R
CTNS
ADRA2A
CTNND1
CTSK
GPBAR1
CTSL
CYLD
CYP2C19
CYP2J2
CYP11A1
CYP26A1
CYP27A1
DGKQ
DAXX
DCN
DEFB4A
DES
DHFR
DIAPH1
SEPTIN1
DLG1

DNASE1
DNTT
DOCK3
DRD2
JAG1
DSC1
DVL1
EEF1A1
EGR2
AHR
EIF2S3
UBR1
EIF4EBP1
SERPINB1
ELAVL4
PIKFYVE
KLHDC8B
CTTN
SLC29A1
EPOR
ERBB2
ERCC1
ERG
ALAD
ETFA
ABCD1
F3
ALDH1A1
FABP7
FANCG
FASN
STOX1
MS4A2
FCGR2A
FES
FGF9
FGF13
FGFR1
FGFR4
FHL2
CARD8
MSRB2
ATF6
RAB21
KDM1A
SETX
ZNF423
KIF1B
MAPK8IP3
FLNB
SEPTIN8
UBXN4
ATG4B
NUP160
FMR1
WASHC4
SIRT5
GRIP1
HEY2
MACF1
PADI4
DDAH1

SNHG1
TMEFF2
RAB38
ALOX12
ALOX5AP
PRPF6
FSHR
FTH1
ABL1
GAST
ACKR1
EBF3
G6PD
GABBR1
PTF1A
GAD2
FBXO7
TXN2
GALNS
SGMS1
ASPM
LRP10
GART
FGF21
MSTN
GFRA1
GHR
GHSR
TPK1
SND1
B3GAT1
DKK3
DKK2
IL37
VPS4A
COQ2
TNFRSF21
GLI1
GNAI1
GNB3
GOLGA2
GOLGA4
ANG
C20orf203
SREK1IP1
GPX4
ANK3
GRIA4
TMEM230
SLC25A4
DROSHA
GRM1
GRM3
CXCL2
GSK3A
BLNK
NPC1L1
GUSB
GYPE
PSAT1
UBQLN2
HARS1

HBG2
HCK
HDC
HIP1
HLA-DQA1
HLA-DQB1
HLA-DRA
HLA-G
HMBS
HMGB2
FOXA2
HNF4A
HNRNPA2B1
HNRNPC
HNRNPK
HSD17B4
BIRC3
HSPA2
XIAP
HSP90AB1
DNAJB1
TNC
IARS1
IRF8
IDH1
IFIT3
IFNAR1
IGBP1
AMIGO2
IGFBP1
IGFBP5
IGHG3
FASLG
IL4R
IL7
IL12B
INSRR
IRAK1
IREB2
IRF7
ITGAV
ITGB3
ITPR2
JAG2
JAK2
JUNB
NHLRC2
KCNMA1
KCNQ1
KIR2DL2
KLKB1
KLRC1
KIF11
ARG2
KRT14
KRT18
LIN28B
LAMC1
LBR
LCAT
LCK
LCT

LDHA
LEPR
LIFR
LOXL1
LRPAP1
LTC4S
LTF
LYN
MIRLET7D
MIR10A
MIR122
MIR127
MIR134
MIR139
MIR140
MIR15A
MIR181A2
MIR186
MIR191
MIR19B1
MIR20A
MIR210
MIR221
MIR223
MIR27A
MIR30B
MIR30E
MIR31
MIR34C
MIR9-1
MIR93
M6PR
ARRB1
MARCKS
SMAD1
SMAD7
MAG
MAP1A
MAP1B
MAT1A
MAT2A
MAZ
MC1R
MCM2
MDH1
MDH2
ASAH1
MID1
CXCL9
ATXN3
NR3C2
MMP7
MMP8
MMP13
MOG
MPI
MRC1
MRE11
MIR133B
MIR335
MIR338
MIR346

MIR369
ASPA
MSR1
MSRA
NUDT1
MTNR1B
MTRR
MMUT
MUTYH
MX1
SERPINC1
MYH9
NDP
NDUFA6
NDUFA9
ATIC
NDUFB8
NEDD4
SEPTIN2
NEU1
NFKB2
NME1
NQO2
YBX1
NT5E
OAT
MIR361
MIR377
BDNF-AS
OMP
OPA1
ACO2
OSM
ALDH7A1
OXA1L
OXT
NOX4
PAK3
PCA3
PAX6
F11R
PC
SAR1B
IRAK4
NT5C3A
CD320
TLR8
HOOK1
PPME1
DCDC2
PDGFB
TDP2
ENPP2
PDYN
MSRB1
INPP5K
PENK
PFKFB3
ATP5PF
PGR
ATP6V1B2
SERPINE2

SERPINI1
PIK3C3
ATP6V1E1
PIK3R1
PIK3R2
PITX2
PLAT
PLAUR
LRP1B
PLEK
PLP1
PML
PMM2
PMP22
ATP5PO
POLG
POR
TOLLIP
CCHCR1
POU3F4
UGT1A1
PDP1
PPIB
TET2
NDE1
QRICH1
CASZ1
PPL
PPP1CB
TAPBPL
PPP2R1A
FBXW7
PPT1
MEG3
MIR429
PRG2
SLC30A10
PRKACA
PRKACB
VAC14
PRKAR1B
OGDHL
CDK5RAP2
TDP1
PRKCD
PRKCE
ADCY10
SELENOS
APOM
MAP2K3
PROC
BTNL2
PARD3
KLK7
KLK10
DPYSL5
ANKS1B
PSMB2
MCCC1
ARNTL2
DUSP22
MFF

PSMD2
PSMD3
CAMK1D
PSMD7
RTN4
PSMD9
PTCH1
PTGDR
PTGER2
MIR431
MIR485
MIR497
MTUS1
HECW2
SORCS2
PTN
EPG5
BAK1
PTPN11
LSM2
PTPN13
PURA
JAM2
RAB27A
RAC2
RAD23B
RAF1
RAN
RASA1
RB1
RET
RHD
RHO
BCL6
BRD2
HPSE2
PROK2
RORA
RPA1
BCR
RPL13
RPL15
RPS3A
RPS23
BDKRB2
S100A8
SAA2
SCN1A
BGN
CCL1
CCL8
CCL20
CCL21
BID
SELE
GOLPH3
BLM
MAP2K4
SRSF1
SRSF5
SRSF6
NOC3L

TRA2B
SFTPC
NMNAT1
P2RY12
AD6
BMP1
PMEL
SLC1A1
SLC5A2
SLC6A1
NCF1
SFTPA1
SLC8A1
SLC10A2
SLC11A1
SLC16A1
SLC18A1
SLC22A2
SLC22A5
SMPD2
SNRNP70
SOD3
SOS1
SOS2
SOX3
SOX5
SP4
SRF
SRM
SSTR2
SSTR3
ST2
ST14
STAR
STIM1
STK11
STX1A
STXBP1
SUOX
ABCC8
VAMP1
VAMP2
TACR2
MAP3K7
TARBP2
TBX2
ELOC
TCF4
MIR590
TCN2
BTK
TCP1
TDO2
TERC
TERF1
TERF2
TGFBI
THBS1
THBS4
THOP1
THRA
TIMP3

C1QBP
TKT
TLR3
TSPAN7
TNFAIP6
TNNI3
TNR
TP53BP1
TPM1
TRAF2
TRH
C4BPA
TSC1
TSG101
C5
TTN
TUBA4A
TWIST1
UBA52
UBC
UBE2A
UBE2D2
UBE2V1
UBE3A
C9
UCP2
UGT1A
UQCRC1
EZR
WARS1
WEE1
WNT5A
WT1
CFAP410
CACNA1C
ZNF224
FZD5
SCG2
SLC25A20
MAPKAP1
DEK
DDX39B
PAGR1
GTDC1
NARS2
FZD3
CAMKMT
ZC3H14
PSCA
PANK2
TM2D3
WDR26
DNAJC5
ADAM12
FGF23
AD5
PABPN1
SLC7A5
RNF146
NRIP1
DYSF
BAP1

EOMES
H4C13
TMEM175
PHF6
GNPAT
MAP1LC3A
FAM126A
IRS4
MFSD2A
LINGO1
GAS7
MAPKAPK5
DEGS1
KMO
PRKRA
TNFSF11
RUNX1
PDE8B
AOC3
NUMB
SOCS1
B3GALT4
SNX3
ADAM19
GBF1
RAB11A
TNFRSF6B
SOCS2
CCKAR
NAE1
KRIT1
RAB29
NAV2
SELENBP1
NAT1
ARTN
LRSAM1
CLDN1
USP2
CCNE2
RABEP1
ATG12
HGS
CHRDL1
CD5L
RAB11B
AIMP1
CD19
MS4A1
CD163
SLIT2
MYOCD
NRXN1
CD28
CYP7B1
HOMER2
STX8
SCARB2
BCAR1
SNCAIP
RGS6
SH3PXD2A

CD63
SDC3
CD81
PIEZO1
BMS1
TOMM20
KIAA0319
TECPR2
RBM8A
CDC25B
CDC25C
HS3ST1
MED12
CCS
HNRNPDL
